# Pd–Pd/PdO as active sites on intercalated graphene oxide modified by diaminobenzene: fabrication, catalysis properties, synergistic effects, and catalytic mechanism†

**DOI:** 10.1039/d2ra00658h

**Published:** 2022-03-18

**Authors:** Zihan Li, Erran Song, Ruirui Ren, Wuduo Zhao, Tiesheng Li, Minghua Liu, Yangjie Wu

**Affiliations:** College of Chemistry and Molecular Engineering, Zhengzhou University Kexuedadao 100 Zhengzhou 450001 P. R. China lts34@zzu.edu.cn (+)86-371-67766667; Henan Institute of Advanced Technology, Zhengzhou University Kexuedadao 100 Zhengzhou 450001 Henan Province P. R. China; Beijing National Laboratory for Molecular Science, Institute of Chemistry, Chinese Academy of Sciences Zhongguancun North First Street 2 Beijing 100190 P. R. China

## Abstract

Pd–Pd/PdO nanoclusters well dispersed on intercalated graphene oxide (GO) (denoted as GO@PPD–Pd) were prepared and characterized. GO@PPD–Pd exhibited high catalytic activity (a TOF value of 60 705 h^−1^) during the Suzuki coupling reaction, and it could be reused at least 6 times. The real active centre was Pd(200)–Pd(200)/PdO(110, 102). A change in the Pd facets on the surface of PdO was a key factor leading to deactivation, and the aggregation and loss of active centres was also another important reason. The catalytic mechanism involved heterogeneous catalysis, showing that the catalytic processes occurred at the interface, including substrate adsorption, intermediate formation, and product desorption. The real active centres showed enhanced negative charge due to the transfer of electrons from the carrier and ligands, which could effectively promote the oxidative addition reaction, and Pd(200) and the heteroconjugated Pd/PdO interface generated *in situ* also participated in the coupling process, synergistically boosting activity. Developed GO@PPD–Pd was a viable heterogeneous catalyst that may have practical applications owing to its easy synthesis and stability, and this synergistic approach can be utilized to develop other transition-metal catalysts.

## Abstract 2

Pd(200) and the Pd(200)/PdO(102, 110) interface generated *in situ* participated in coupling reactions *via* a synergistic effect, boosting the catalytic activity to a high level.

## Graphical abstract

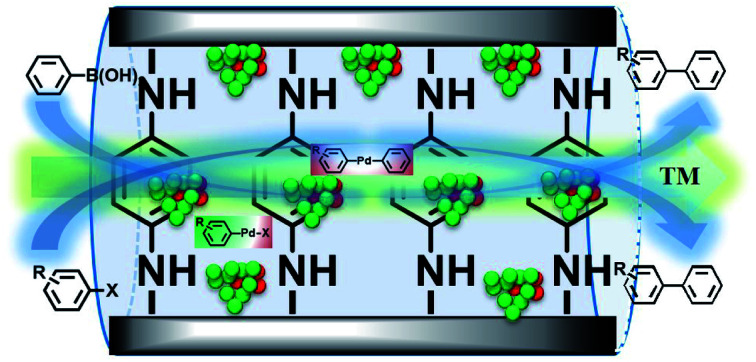

## 1. Introduction

Palladium catalysts are widely used during C–C coupling reactions, such as Suzuki,^1^ Heck,^2^ and Sonogashira^3^ reactions, which have been extensively studied.^4,5^ The Suzuki–Miyaura cross-coupling reaction can be used to synthesize numerous organic compounds, including pharmaceuticals, herbicides, natural products, intermediates, and conductive polymers,^6–9^ and therefore it is considered to be one of the most effective transformations.^10^ As we know, homogeneous palladium catalysts exhibit high selectivity and good dispersion in solvents, and they can be easily synthesized. However, they are difficult to separate from reaction mixtures, restricting their use in industrial production due to high costs and potential heavy-metal pollution.^11–13^ Therefore, there is an urgent need to develop more effective and environmentally friendly catalysts.

Heterogeneous catalysts with high activity have aroused the interest of researchers in recent years, and they can be reused following simple filtration and centrifugation.^14–17^ The catalytic properties of a heterogeneous catalyst depend on the active composition, the structure of the catalyst, and the used support.^18^ Palladium catalysts supported on solid materials have been reported, with supports such as carbon materials,^19–21^ mesoporous silica materials,^22–24^ polymers,^25–27^ metal–organic frameworks,^28^ and metallic oxides.^29–35^ Among the different supports used, graphene oxide (GO) has been the subject of intense interest because of its inimitable structural and electronic properties.^36–40^ Additionally, the abundant functional groups on the surface of GO facilitate the covalent modification of the surface to improve the stability of associated catalysts.^41–43^ Moreover, the two-dimensional structure of graphene oxide facilitates the dispersion of organic substrate molecules and promotes catalytic reactions.^44^ These characteristics lead to a broad development space in areas such as heterogeneous catalysis, sensors, solar cells, and gas storage.^45–49^ Yang *et al.* synthesized polydispersed graphene sheets modified with an amine derivative,^50^ and the amine-functionalized graphene oxide can stabilize metal nanoparticles and distribute them well, avoiding aggregation and thus increasing the catalytic activity.^51–56^ Recently, Ma *et al.* prepared a graphene-based Pd hybrid catalyst modified with DNA (called DNA–G–Pd), which could chelate Pd *via* dative bonding, showing enhanced catalytic activity toward the Suzuki reaction.^57^ In recent years, three-dimensional graphene frameworks with porous structures have become ideal carriers for preparing heterogeneous catalysts, having a large specific surface area, adjustable porosity, controlled active site growth, and high stability.^58–60^ As we know, utilizing a covalent-bond-based structure assisted by ligands acting as linkers is a possible strategy for improving the stability of three-dimensional graphene materials and avoiding the aggregation of nanoparticles.^61–63^ Yuan *et al.* developed a novel way to encapsulate Pd nanoparticles inside a GO framework, using covalent intercalation with benzene-1,4-diboronic acid, resulting in a periodic layered structure and high activity toward the Suzuki coupling reaction.^64^ In the case of using metal oxides as supports, it is still a challenge to obtain Pd–metal oxide stability at the interface due to interconvertibility, while metal oxide surfaces exhibit good selectivity toward different substrates. Strong metal–support interaction can be strongly influenced by reducible oxide supports, and this can also concomitantly boost activity.^29–36^ Considering that a combination of Pd–Pd/PdO with intercalated GO might play a key role in catalysis, it is plausible to postulate what is the most suitable way to enhance catalytic activity and stability in a controlled manner.

Self-assembly is usually used to construct desired structures, offering controllable orientation and thickness, and stable monolayers, allowing the catalytic activity to be enhanced.^65–76^ Therefore, using self-assembly between a suitable ligand and GO to construct layered monolayers is expected to help with the immobilization, stabilization, and good distribution of Pd–metal oxide nanoclusters, offering an ideal catalytic surface for understanding what is truly happening at the interface.

In this work, a simple approach for preparing Pd–metal oxide supported by diamino-modified GO was reported. The catalytic activity, structure of active centres, and catalytic mechanism were investigated in detail, using the Suzuki coupling reaction as a template.

## 2. Experimental

### 2.1 Chemical reagents

All chemical reagents were obtained from different commercial sources and used without further purification.

### 2.2 Characterization

X-ray diffraction (XRD) patterns were obtained using a PAN analytical X-Pert PRO instrument. Fourier-transform infrared spectroscopy (FTIR) analysis was carried out using a BRUKER TENSOR FTIR spectrometer using KBr pellets in the range of 400–4000 cm^−1^ with a resolution of 4 cm^−1^. Raman spectra were obtained using a Thermo Scientific DXR Raman microscope with an excitation laser wavelength of 532 nm. X-ray photoelectron spectroscopy (XPS) analysis was carried out using an ESCALab220i-XL electron spectrometer from VG Scientific with 300 W Al Kα radiation. Scanning electron microscopy (SEM) images were recorded using a Hitachi S-4800 system. Transmission electron microscopy (TEM) images were obtained using a JEM-2100F transmission electron microscope operating at 200 kV. The size distributions of palladium nanoparticles were calculated from representative TEM images at a constant magnification based on 100 randomly selected nanoparticles. The Pd content was measured using ICAP 6000 Series apparatus (Thermo Scientific). *S*_BET_ data from the as-prepared catalyst were obtained based on N_2_ adsorption–desorption data and BET measurements (ASAP2020, Micromeritics, USA). Electrochemical impedance spectra (EIS) were obtained using a three-electrode system (CHI660, CH Instrument, USA). During these experiments, 0.1 M aqueous Na_2_SO_4_ was used as an electrolyte, and a Ni foam electrode fully covered with the as-obtained catalyst was used as the working electrode. Thermogravimetric analysis (TGA) was carried out using an STA 409 PC Thermal Analyzer under a nitrogen atmosphere in the range of 30–800 °C at a heating rate of 10°C min^−1^. ^1^H-NMR and ^13^C-NMR spectra were recorded using a Bruker Avance III 400 MHz spectrometer in CDCl_3_ with tetramethylsilane as an internal standard.

### 2.3 Preparation of amino-modified graphene oxide (GO@PPD)

Firstly, graphene oxide (GO) (100 mg) was dispersed in ethanol (20 mL) in a round-bottom flask *via* ultrasonic treatment for 2 h. Then, *p*-phenylenediamine (PPD, 200 mg) and KOH (200 mg) were added into the above flask. The suspension in the flask was subjected to ultrasonication for 30 min. Finally, the mixture was refluxed at 80 °C for 24 h under vigorous stirring. The resulting solution was subsequently centrifuged and washed several times using absolute ethanol and deionized water to remove unreacted PPD, and the obtained product was dried in a vacuum oven at 40 °C for 12 h.

### 2.4 Preparation of palladium catalyst derivatives supported on graphene oxide modified with diaminobenzene (GO@PPD–Pd)

Li_2_PdCl_4_ (0.1 M) was prepared *via* mixing 177 mg of PdCl_2_, 85 mg of LiCl, and 10 mL of anhydrous methyl alcohol in an Erlenmeyer flask, followed by stirring at room temperature for 24 h. GO@PPD (100 mg) was dispersed in anhydrous methyl alcohol (20 mL) and sonicated for 1 h. Then, avoiding the use of any supplementary reductants and stabilizers, Li_2_PdCl_4_ (200 μL, 100 μL, or 300 μL) was directly added into the above mixture under continuous stirring at 40 °C for 24 h to prepare GO@PPD–Pd, GO@PPD–Pd1, and GO@PPD–Pd2, respectively. The mixture was cooled to room temperature, centrifuged, and washed several times with dichloromethane, absolute ethanol, and alcohol. The obtained product was dried in a vacuum oven at 40 °C for 12 h. For comprehensive comparison, GO@Pd was also synthesized using the same procedure without the addition of PPD. The Pd content levels in the as-prepared GO@PPD–Pd, GO@PPD–Pd1, GO@PPD–Pd2, and GO@Pd samples were further determined *via* ICP-AES.

### 2.5 Suzuki coupling reaction catalysed by GO@PPD–Pd

The catalytic activity of the GO@PPD–Pd catalyst was tested based on the Suzuki coupling reaction; 4-bromotoluene (0.25 mmol), phenylboronic acid (0.3 mmol), base (0.5 mmol), and GO@PPD–Pd (1 mg) were added to a reaction tube with 4 mL of solvent. The reaction mixture was carried out in an oil bath under vigorous stirring at a specified temperature. After the completion of the reaction, the GO@PPD–Pd catalyst was separated from the reaction mixture *via* filtration, and the coupling products were separated using a chromatographic column.

### 2.6 Recycling experiments

Recycling experiments were carried out under the above-described conditions. After each run, the used catalyst was filtrated, washed with water, methanol, ethanol, and chloroform several times, and reused for sequential runs.

## 3. Results and discussion

### 3.1 The fabrication route to GO@PPD–Pd

**Scheme 1:**
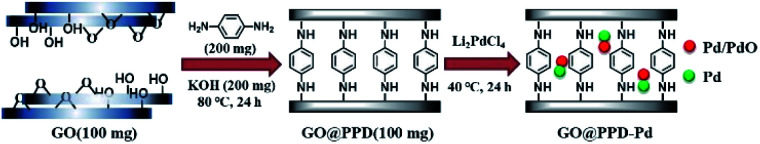
The fabrication route to GO@PPD–Pd.

The fabrication route to the GO@PPD–Pd catalyst is depicted in Scheme 1.

### 3.2 Characterization of GO@PPD–Pd

XRD spectra of GO, GO@PPD, and GO@PPD–Pd were obtained (Fig. S1†). Characteristic peaks at 42.35° and 11.43° were observed, which can be assigned to the (100) plane of the hexagonal structure of carbon and the (001) plane of GO. The two peaks shifted to 43.12° and 10.53° in the case of GO@PPD, showing expanded *d*_001_ interlayer spacing and decreased *d*_100_ interlayer spacing. A broad peak appeared at 25.45° corresponding to the (002) plane of graphite, indicating that partially reduced GO was modified with PPD; in addition, the diffraction peak widened, indicating a decrease in the integrity of the crystal structure and an increase in disorder. The presence of Pd immobilized on GO@PPD led to the characteristic peak of GO being shifted to 11.20° due to interactions between the graphene oxide layers. Moreover, a new weaker peak at 39.60° corresponding to the (111) plane of Pd appeared, indicating that Pd nanoparticles were formed from Li_2_PdCl_4_ reduced by nitrogen groups, which can be used as reducible agents and stabilizers to help form Pd nanoparticles.^77^

FT-IR spectra were recorded throughout the fabricating process of GO@PPD–Pd, and they are shown in Fig. S2.† Peaks at 3426, 1726, 1622, 1398, 1215, and 1049 cm^−1^ correspond to hydroxyl stretching, carbonyl stretching (Fig. S2,† black line), C–OH vibrations, –CH_2_– stretching, C–O stretching, and epoxy vibrations. After modification with PPD, three distinct peaks at 1562, 1120, and 625 cm^−1^ were observed, which can be assigned to N–H vibrations, C–N stretching, and N–H stretching, which further confirmed the attachment of PPD to the graphene oxide nanosheets. Meanwhile, the peak at 1726 cm^−1^ disappeared, showing that GO was reduced. In the case of GO@PPD–Pd, no obvious changes were observed compared with GO@PPD.

Raman spectroscopy is usually used to investigate the structural disorder of GO.^78^ The two distinct peaks at around 1592 and 1346 cm^−1^ can be assigned to the G and D bands of GO (Fig. S3†), respectively. The D band is related to the disordered vibrations of sp^3^ carbon, while the G band is related to the vibrations of sp^2^ carbon atoms in GO. The *I*_D_/*I*_G_ intensity ratio of GO@PPD (1.04) increased compared to GO (0.96), which reflected that the number of sp^2^ carbon atoms had decreased, and the *I*_D_/*I*_G_ ratio of GO@PPD–Pd(1.01) was lower than that of GO@PPD, indicating that interactions occurred between Pd nanoparticles and amino-modified graphene oxide. The D band position showed almost no change after modification with PPD, and the G band at 1592 cm^−1^ shifted to 1583 cm^−1^, which was ascribed to the reduced GO.^79^

**Fig. 1:**
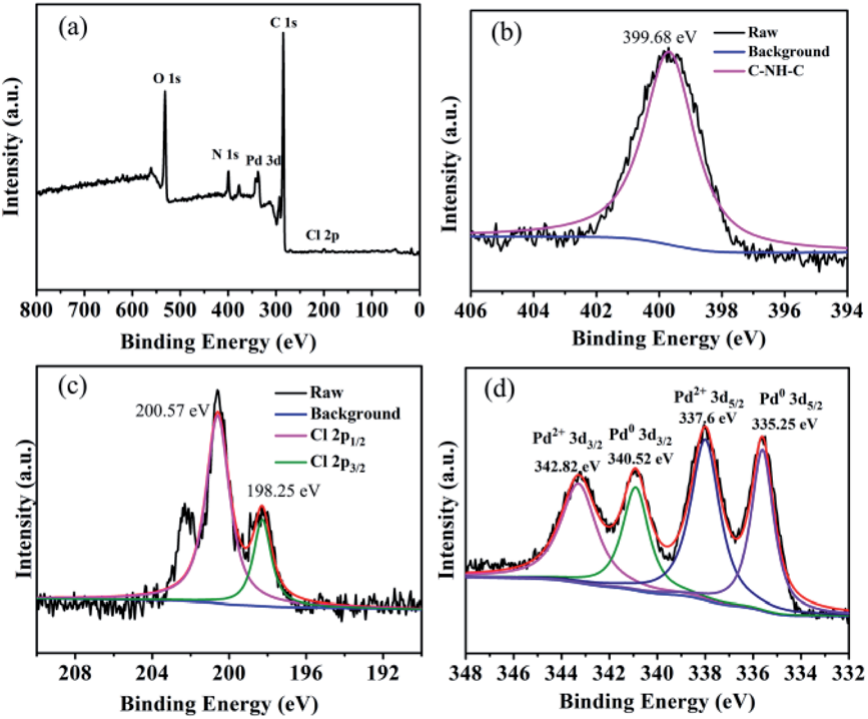
(a) XPS survey spectrum of GO@PPD–Pd, (b) HR-XPS of N 1s, (c) HR-XPS of Cl 2p and (d) HR-XPS of Pd 3d.

XPS spectra were obtained throughout the preparation to explore the surface composition (Fig. 1).^80^ The measured survey spectrum exhibited C, O, N, Cl, and Pd peaks (Fig. 1a), which matched the elemental composition of GO@PPD–Pd. N 1s HR-XPS analysis showed a single peak at a BE of 399.30 eV, corresponding to nitrogen–hydrogen bonds, confirming that PPD molecules were successfully modified onto graphene oxide (Fig. 1b). Pd 3d HR-XPS analysis of GO@PPD–Pd showed distinct peaks from Pd^0^ at 335.25 and 340.52 eV, which could be assigned to Pd^0^ 3d_5/2_ and Pd^0^ 3d_3/2_, respectively, suggesting the existence of partially reduced Pd species in GO@PPD–Pd. Peaks at 200.57 eV and 198.25 eV were attributed to Cl 2p_1/2_ and Cl 2p_3/2_, showing the presence of Cl coordinated with palladium in the catalyst. Two characteristic peaks at 342.82 and 337.6 eV were assigned to Pd^2+^ 3d_3/2_ and Pd^2+^ 3d_5/2_ (Fig. 1d). The XPS results confirmed the simultaneous existence of palladium nanoparticles and divalent palladium in GO@PPD–Pd.

The morphologies of GO, GO@PPD, and GO@PPD–Pd were studied *via* SEM, as shown in Fig. S4.† A large number of folded and layer-like GO structures were present (Fig. S4a†), and the morphology showed a relatively neat sheet-like structure after modification with PPD (Fig. S4b†). The SEM image of as-synthesized GO@PPD–Pd shows the good distribution of Pd nanoparticles, indicating that GO@PPD–Pd was prepared (Fig. S4c†).

**Fig. 2:**
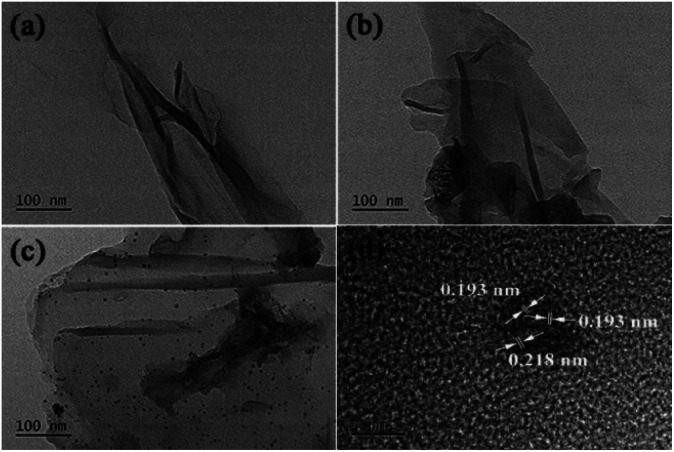
TEM images of (a) GO, (b) GO@PPD, and (c) GO@PPD–Pd, and (d) a HRTEM image of GO@PPD–Pd.

The morphology and structure of GO@PPD–Pd were further investigated *via* TEM analysis. As shown in Fig. 2, layered structures of GO were confirmed (Fig. 2a), and similar layered structures were also observed after PPD modification (Fig. 2b), demonstrating the morphology of GO. Well-dispersed nanoparticles were observed (Fig. 2c) after Pd modification. The lattice spacings of the particles in Fig. 2c are 0.193 nm and 0.218 nm based on high-resolution imaging (Fig. 2d), corresponding to Pd(200) crystalline planes and Pd/PdO (111: 0.224 nm; 110: 0.211 nm; 102: 0.20 nm) polycrystalline planes (solid solution). The lattice spacing (0.218 nm) is between the distances of Pd (111: 0.224 nm) and PdO (102: 0.200 nm; 110: 0.211 nm),^30^ suggesting that a solid solution containing Pd(200) and Pd(111)/PdO(110) clusters is immobilized on GO@PPD–Pd, and Pd(111)/PdO(110) planes may be regarded as sites serving as templates for the formation of Pd(200).^31,81^

**Fig. 3:**
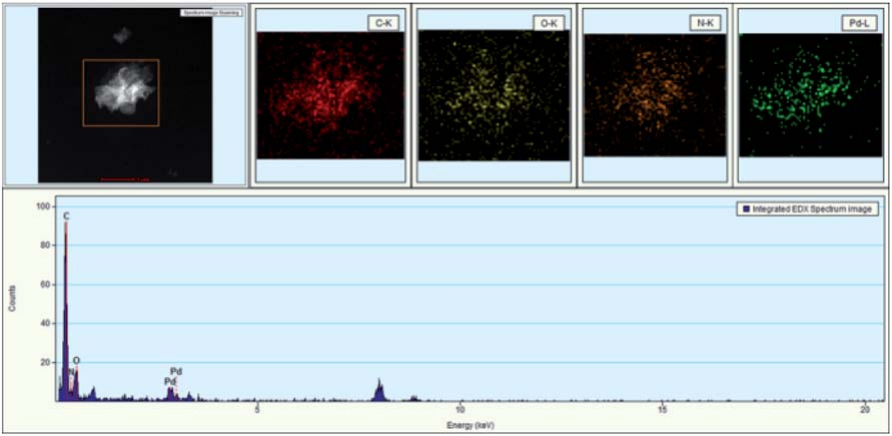
TEM images and EDX analysis of GO@PPD–Pd.

Meanwhile, EDX (energy dispersive X-ray) images of GO@PPD–Pd were also obtained (Fig. 3); carbon, oxygen, nitrogen, and palladium elements were present on the surface of GO@PPD–Pd, suggesting that the material had been successfully modified with ligands and Pd. In addition, the good dispersion of palladium on the modified GO could strengthen the interactions between amino groups, oxygen atoms, palladium, and palladium oxide species.

Brunauer–Emmett–Teller (BET) analysis of the catalyst was performed (Fig. S5†). The adsorption isotherms are presented in Fig. S5a,† clearly indicating that the isotherm of graphene oxide was type IV with a H4 hysteresis loop, while the isotherms of GO@PPD and GO@PPD–Pd were type IV with a H3 hysteresis loop. This is characteristic of mesoporous materials.^82^ BJH analysis (Fig. S5b†) exhibited that the size of the pores was in the range from 2–10 nm, which is consistent with a mesoporous material. In addition, the *S*_BET_ and pore size values of GO, GO@PPD, and GO@PPD–Pd are listed in Table S1.† Obviously, changes in the surface area and pore size were induced by the chemical modification of the surface of GO.

Electrochemical impedance spectroscopy (EIS) analysis of GO, GO@PPD, and GO@PPD–Pd was carried out, as shown in Fig. S6.† The arc radius represents the electrochemical reaction impedance, and the smaller the arc radius, the faster the electron transfer efficiency. Notably, the arc radius showed an obvious decrease as the fabrication process proceeded, predicting that GO@PPD–Pd has superior charge transfer abilities, which could help improve its catalytic activity.

The thermal behaviour as the catalyst fabrication process processed was further analysed using thermogravimetric analysis (TGA). As shown in Fig. S7,† weight loss from GO below 100 °C occurred because trapped moisture was removed, and significant weight loss was observed in the range of 180–240 °C because of the decomposition of groups containing oxygen, giving CO_2_, CO, and steam.^83,84^ However, the slow decomposition above 240 °C was attributed to the pyrolysis of the carbon skeleton (Fig. S7,† black line). The TGA curves of GO@PPD and GO@PPD–Pd showed a slow increase in decomposition with an increase in temperature, implying that the catalyst had higher thermal stability due to the introduction of diamino groups and immobilized Pd (Fig. S7,† red and blue lines).

The characterization results obtained above show that the diaminobenzene-modified graphene-oxide–supported metal palladium catalyst (GO@PPD–Pd) was successfully prepared.

### 3.3 Catalytic properties

#### 3.3.1 Catalytic properties of GO@PPD–Pd

The catalytic performance of GO@PPD–Pd was explored (Table S2†). Initially, the effects of various solvents were investigated; water as a solvent gave a low yield (entry 1), methyl alcohol as a solvent provided a moderate yield (entry 2), and the use of ethanol provided an excellent yield (entry 3). For reasons associated with green chemistry and environmental friendliness, a series of different proportions of water with ethanol was studied; the yields were 92% and 99% (at 70 °C for 30 min with K_2_CO_3_ as the base) in 1 : 1 and 1 : 2 aqueous ethanol solvents, respectively (entries 8 and 9), and there were lower yields with other solvents (entries 4–7 and 10), suggesting that a specific ratio of ethanol to water was conducive to boosting the catalytic reaction.^5^ Then, different bases were studied (entries 11–15), and K_2_CO_3_ was selected due to its economy and high yield (entry 11). Furthermore, the yield decreased upon decreasing the temperature and time (entries 16–21), and only 76% yield was obtained at 30 °C after 30 min (entry 19). Considering the maximum yield and economic factors, the optimized reaction conditions were set to 60 °C for 20 min (entry 20). Additionally, when the amount of substrate was increased from 0.5 mmol to 1 mmol (entries 22 and 23), a yield of 86% with a higher TOF (60 705 h^−1^) was obtained (entry 23), implying excellent activity arising from the good dispersion of stabilized Pd nanoparticles on the modified graphene oxide.

#### 3.3.2 Screening the substrate scope

Further extended experiments involving GO@PPD–Pd were carried out to screen the substrate scope. As shown in Table S3,† good yields could be obtained upon the use of aryl iodides (entries 1–2). In the case of aryl bromides, both *para*-substituted and *meta*-substituted electron-withdrawing and electron-donating groups could give high yields (entries 3–10), but not *ortho*-substituted examples, due to steric effects (entries 11). However, no product was observed when using chlorobenzene derivatives containing either electron-donating or electron-withdrawing groups (entries 12–14) due to the higher bond energy of the C–Cl bond.

#### 3.3.3 Influence of the Pd content on the catalytic performance

To determine the influence of Pd loading on the catalytic activity, reactions catalysed by GO@PPD–Pd, GO@PPD–Pd1, and GO@PPD–Pd2 with different Pd content levels and by GO@Pd without ligands were carried out. The Pd content levels are shown in Table S4.† It was clear that the catalytic activity of GO@PPD–Pd was significantly improved in a promising way compared to GO@Pd, which is attributed to the intercalated structure.^52^ Considering the yields and TOF values, GO@PPD–Pd was used for subsequent research. In addition, TGA was performed to analyse the thermal stability (Fig. S8†). Since the temperature during the Suzuki coupling reaction is generally lower than 100 °C, thermogravimetric analysis curves of the four catalysts from 30–100 °C were obtained. The results showed that GO@Pd offered higher thermal stability below 100 °C. However, the yield was only 5%. Based on the optimum temperature (60 °C), GO@PPD–Pd provided higher thermal stability under the catalytic reaction conditions.

#### 3.3.4 Comparison experiments

Control experiments were carried out to elucidate the effects of GO and structure on the catalytic performance (Table S5†). No desired product was obtained with GO, PPD, or GO@PPD (entries 1–3). The influence of GO on the catalytic properties was explored (entries 4–7). 76% yield was obtained using Li_2_PdCl_4_ (entry 4), and only 64% yield was obtained using a mixture of GO and Li_2_PdCl_4_ as the catalyst (entry 5). PPD + Li_2_PdCl_4_ was also tested, obtaining only a trace yield (entry 6) due to the presence of PPD as a toxic reagent that could cover active sites. 38% yield was obtained using GO@PPD + Li_2_PdCl_4_ (entry 7). Compared with Si@PPD–Pd, GO@PPD–Pd exhibited a higher yield with a high TOF value (entries 8 and 9), predicting that GO also plays an important role in determining the catalytic activity. The results above indicated that amine groups introduced onto the surface of GO could help to disperse active centres and increase the activity through electron donation.

To consider the effects of ligand structure, derivatives were used to modify GO, and their catalytic properties were investigated, as shown in Table S6.† When *m*-phenylenediamine or other derivatives were used, lower TOF values were obtained (entries 2 and 4–8). However, in the case of *o*-phenylenediamine, a higher TOF value was obtained (entry 3), indicating that the effects of the structures of the ligands used, such as electronic effects and steric effects, could efficiently influence the activity.

The catalytic performance of GO@PPD–Pd is compared with similar previously reported palladium catalysts in Table S7.†

#### 3.3.5 Recycling experiments

**Fig. 4:**
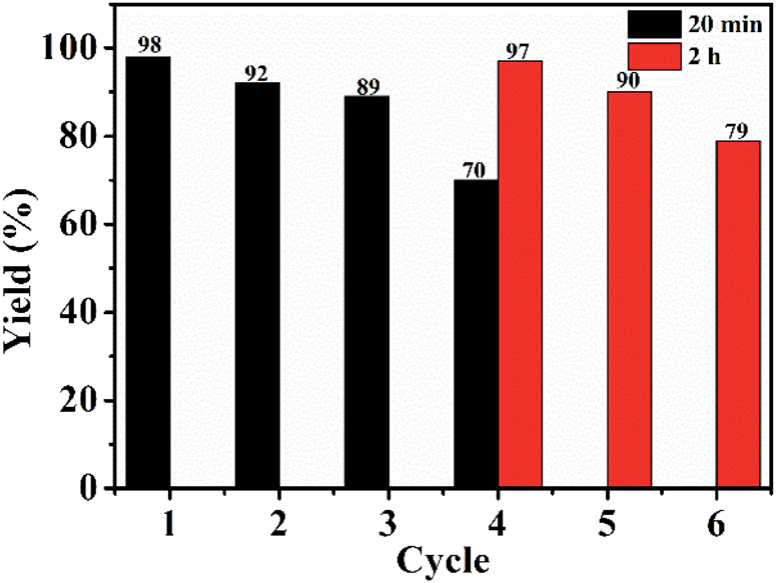
Recycling of GO@PPD–Pd.

To investigate the recyclability of GO@PPD–Pd, experiments were carried out under standard reaction conditions.^85^ As shown in Fig. 4, GO@PPD–Pd displayed reasonable recyclability; although the isolated yield decreased during the fourth cycle, high yields could be obtained from the fourth, fifth, sixth, and seven cycles *via* extending the reaction time to 2 h (red bars).

Almost all supported heterogeneous catalysts show significant loss of activity after reuse. Elucidating the deactivation mechanism is important for enhancing the activity and reusability. Therefore, SEM and TEM analysis were used to investigate the reasons for deactivation.

**Fig. 5:**
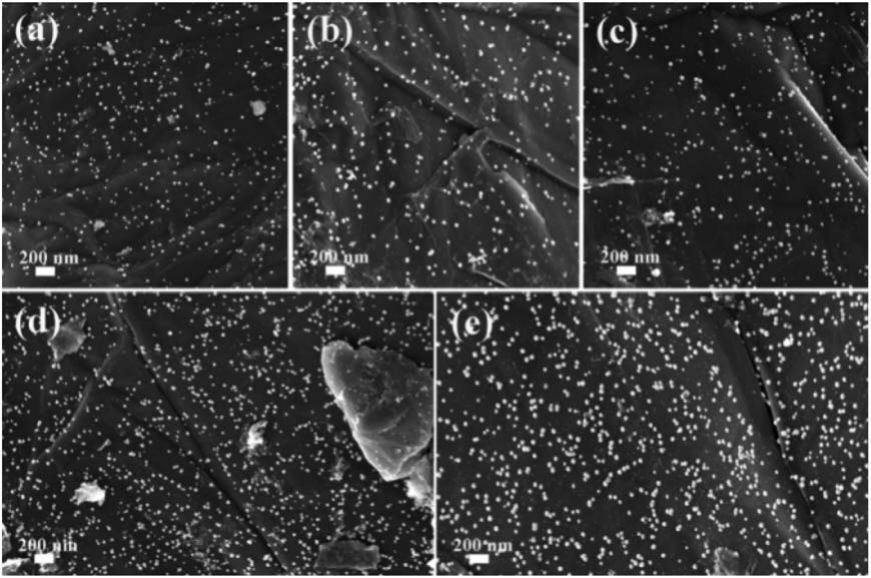
SEM images of GO@PPD–Pd during the reaction and after recycling: (a) 0 min, (b) 10 min, (c) 20 min, (d) 4th run, and (e) 6th run.

To further investigate the morphologies of GO@PPD–Pd before and after reuse, SEM images were obtained (Fig. 5). The results showed that the morphology was preserved during the catalytic process and after recycling (Fig. 5a–e). However, there were different degrees of active Pd nanoparticle aggregation (Fig. 5e), which might be a possible cause of deactivation.

**Fig. 6:**
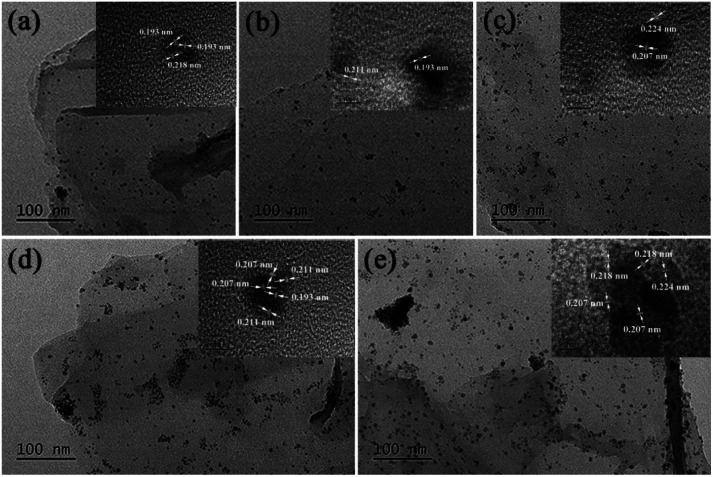
TEM images of GO@PPD–Pd during the reaction and after recycling: (a) 0 min, (b) 10 min, (c) 20 min, (d) 4th run, and (e) 6th run.

TEM images of GO@PPD–Pd obtained during the catalytic process were also investigated, as shown in Fig. 6. Nanoparticles were dispersed without aggregation before catalysis (Fig. 6a), and a HR-TEM image of a chosen nanocluster illustrates Pd(200) and Pd(200)/PdO(102, 110) with a phase boundary, as shown in Fig. 2d.^81^ It was obvious that two discrete nanoclusters of Pd(200) and PdO(110) were observed after 10 min (Fig. 6b), predicting that the active species might be Pd(200). The existence of Pd(200) and Pd(200)/PdO(110, 102) with a phase boundary suggests that charge transfer may be an important factor responsible for the catalytic activity.^86^ The Pd–PdO interface as an active site was also observed after 20 min (Fig. 6c), meaning that the Pd(200) facet and Pd(200)/PdO(110) interface played a vital role in the catalytic process.^34^ A slightly agglomerated cluster composed of Pd(111), the main domain, and PdO(110, 102) with a phase boundary was also observed after the fourth run, with the Pd(200) facet changing into Pd(111), suggesting that the intrinsic activity of Pd(0) with suitable facets was important for enhancing the activity. Upon moving to the sixth recycling cycle, large-scale agglomeration occurred, with Pd(111) and Pd(200)/PdO(110, 102) having segregated distortion planes outside of the cluster, and distinct domains with different orientations were formed, indicating the destruction of the nanocluster (Fig. 6e). The results demonstrated that a change in the Pd facets and the destruction of active centres were the main reasons for deactivation.^87^ It was also evident that the co-existence of stabilized Pd/PdO and Pd(0) phases on intercalated GO could efficiently enhance the activity.^88^

**Fig. 7:**
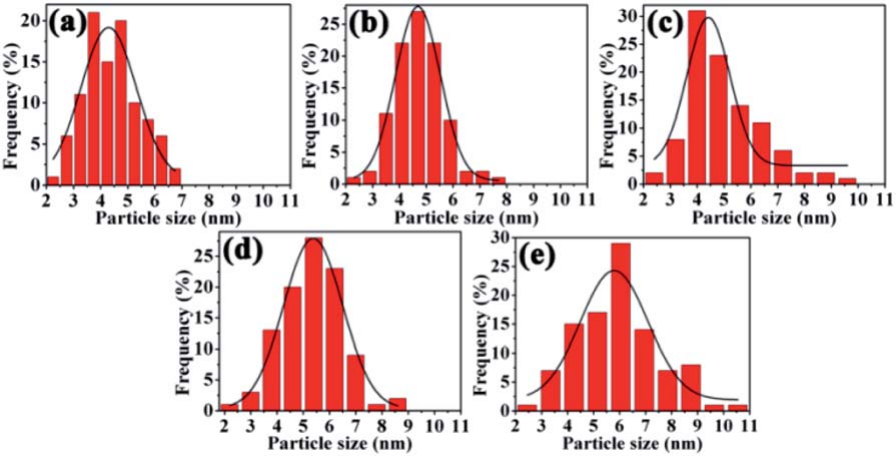
Histograms showing the diameters of Pd nanoparticles during the reaction and after recycling, determined from a sample population of 100: (a) 0 min (4.40 nm); (b) 10 min (4.74 nm); (c) 20 min (5.01 nm); (d) 4th run (5.32 nm); and (e) 6th run (5.92 nm).

Meanwhile, the size distributions of the nanoclusters were calculated, as shown in Fig. 7. The average size of palladium nanoparticles changed from 4.40 to 5.01 nm after 20 min (Fig. 7a–c), and average sizes of 5.32 and 5.92 nm were observed after the fourth and sixth runs, respectively (Fig. 7d and e). The results implied that the aggregation of Pd–Pd/PdO active centres was a key factor explaining the deactivation. The retained Pd content levels were measured to be 3.07 × 10^−5^ mmol mg^−1^ after the fourth cycle and 8.56 × 10^−6^ mmol mg^−1^ after the sixth cycle, indicating the loss of Pd compared with a fresh sample (4.25 × 10^−5^ mmol mg^−1^). The results showed that deactivation could mainly be attributed to the structure of the active centres, including changes to the Pd facet and the aggregation and loss of active species during recycling, which led to the loss of activity.

### 3.4 Investigation into the catalytic mechanism

#### 3.4.1 Hot filtration experiment

**Fig. 8:**
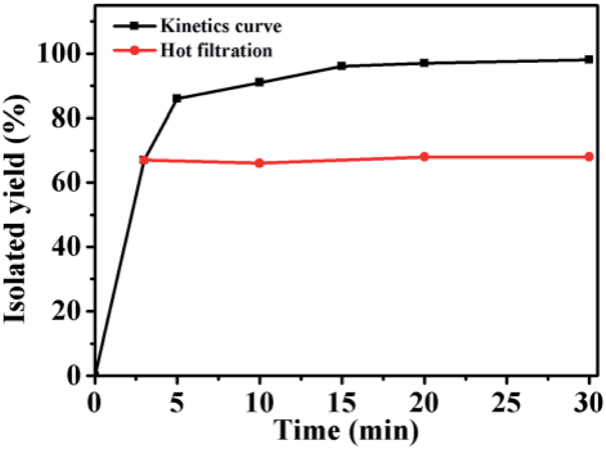
Hot filtration testing involving GO@PPD–Pd.

The catalytic process was studied through kinetics analysis, as shown in Fig. 8. In the first 3 min of the reaction, the yield increased rapidly with reaction time, but it then increased more slowly. The yield of the reaction after 20 min reached 97% (Fig. 8, black line). In order to investigate whether Pd leached out during the catalytic process, a hot filtration experiment was conducted. The catalyst was filtered out after 3 min, and the remaining residual solvent was then used to continue the reaction (Fig. 8, red line). The yield basically stopped increasing as the reaction time increased, indicating that there was almost no Pd leaching into the solution.

In order to distinguish between heterogeneous and homogeneous catalysis and to investigate where the catalytic reaction occurred, poisoning experiments were carried out (Table S8†).^89^ As is known, a poisoning reagent can effectively form bonds with the metal centres of a heterogeneous catalyst, resulting in a loss of activity. The activity of GO@PPD–Pd obviously decreased upon the addition of 0.5 equivalents of 2,2′-dipyridyl, which is an effective poisoning reagent (entry 2). However, when thiophene was added to the reaction mixture, the activity obviously decreased, but the catalyst was not completely deactivated. A possible reason was that thiophene could not coordinate with palladium completely, indicating that catalytic processes occurred at the interface. Combined with the hot filtration experiment results, the existence of a heterogeneous catalytic process can be confirmed.

#### 3.4.2 Electrochemical impedance spectroscopy (EIS), Raman, and X-ray diffraction (XRD) analysis during the catalytic process

**Fig. 9:**
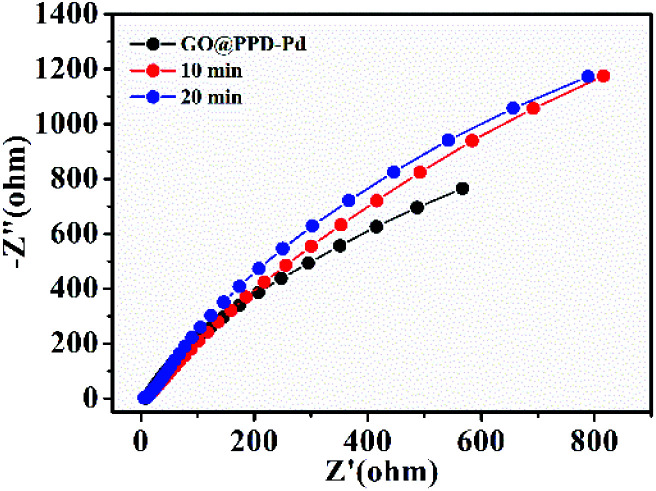
Electrochemical impedance spectra (EIS) of GO@PPD–Pd on Ni foam during the catalytic process.

Furthermore, electrochemical impedance spectroscopy (EIS) testing during the catalytic reaction was carried out (Fig. 9). The arc radius showed a tendency to increase during the catalytic reaction process, suggesting the aggregation of nanoparticles and the adsorption of substrates and product, which could reduce the electrical conductivity and inhibit charge transfer.

**Fig. 10:**
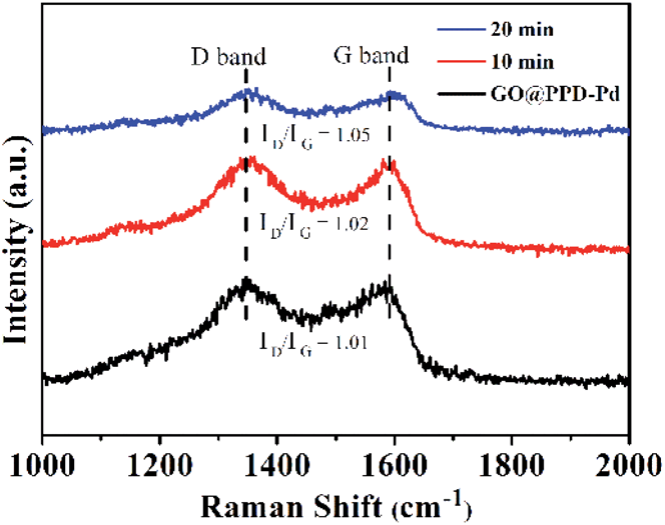
Raman spectra of GO@PPD–Pd during the catalytic process.

Raman spectra of GO@PPD–Pd during the catalytic process were recorded (Fig. 10). The distinct D and G bands showed no shifts, suggesting high stability during catalysis. However, the *I*_D_/*I*_G_ intensity ratio slightly increased with an increase in the reaction time, which was ascribed to micro-changes in the layered GO structure.

**Fig. 11:**
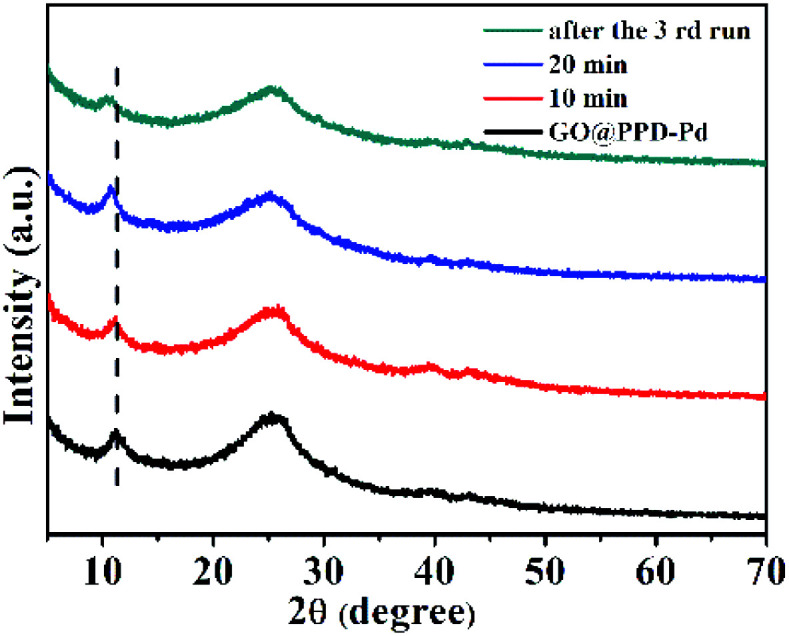
XRD patterns of GO@PPD–Pd during the catalytic process and after the 3rd reaction cycle.

XRD patterns of GO@PPD–Pd during the catalytic process and after the third run were recorded (Fig. 11). The characteristic peak of GO at 2*θ* ≈ 11.20° shifted to a lower angle of 11.17° after 10 min, to 10.70° after 20 min, and to 10.36° after the third run, which indicated that the interlayer distance became larger due to the slight aggregation of palladium or substrates in the layered graphene oxide structure.

#### 3.4.3 X-ray photoelectron spectroscopy (XPS) analysis

**Fig. 12:**
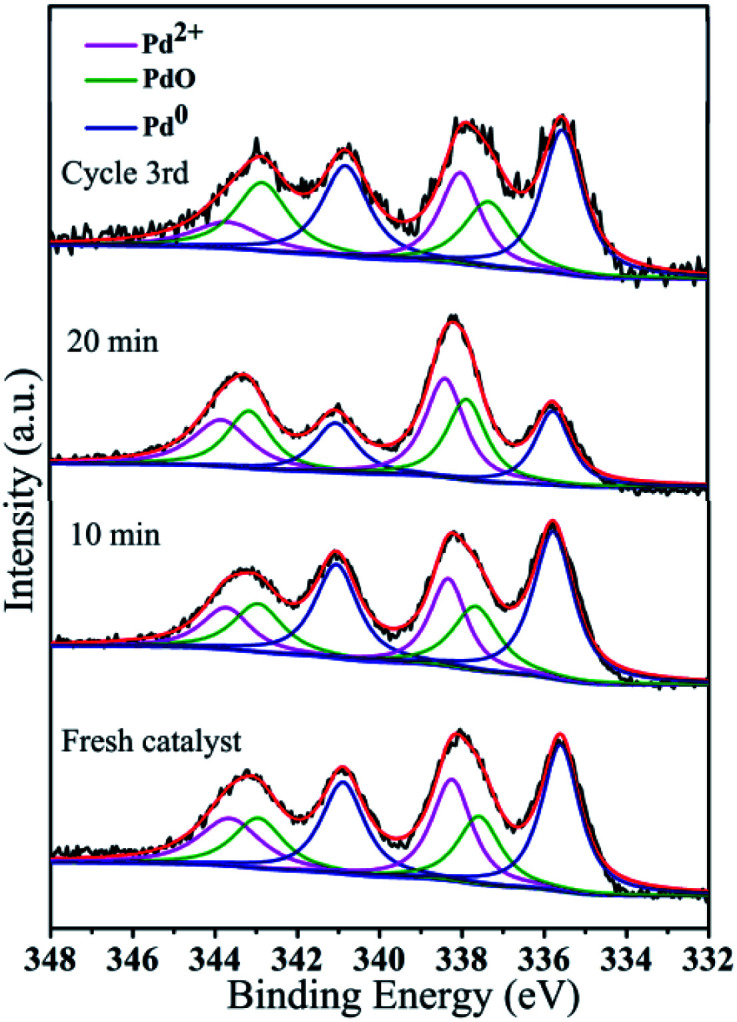
Pd 3d HR-XPS spectra during the catalytic process and after reuse: fresh catalyst, 10 min, 20 min, and cycle 3rd.

XPS is an effective tool for detecting changes in the valence state of a metal during a catalytic reaction.^90^ As shown in Fig. 12, HR-XPS analysis of the active nanoclusters in GO@PPD–Pd during the reaction and after recycling was carried out. For the fresh catalyst, the peak positions and assignments of Pd, Pd^2+^, and PdO are as shown in Fig. 12 (Pd, blue line; Pd^2+^, green line; PdO, pink line).^34,86^ XPS can be utilized for semi-quantitative elemental analysis; the area of the peak can reflect the content or relative concentration of atoms. Therefore, the PdO(Pd^2+^)/Pd^0^ area ratio of fresh GO@PPD–Pd was 1.40. Compared to the intensity of the PdO(Pd^2+^) peak at 0 min, the intensity of the Pd^0^ peak increased significantly after 10 min, and the PdO(Pd^2+^)/Pd^0^ ratio was 1.10, showing that some of the PdO(Pd^2+^) was reduced during the reaction. This might be related to GO transferring electrons to Pd^2+^*via* ligands, making Pd^0^ more negative, and allowing oxidation addition to proceed. After 20 min, the intensity of the Pd^0^ peak decreased, and the PdO(Pd^2+^)/Pd^0^ ratio was 1.58, indicating that Pd^0^ was oxidized to PdO(Pd^2+^) and validating the presence of an oxidation–reduction cycle, obtaining balance between Pd and PdO(Pd^2+^) as a result of constant exchange during catalysis.^91^ The above analysis suggested that Pd^0^/PdO might be the active centre. After GO@PPD–Pd was recycled a third time (3rd cycle), the PdO(Pd^2+^)/Pd^0^ ratio was 0.85 due to the aggregation and irreversible valence of active centre Pd^0^ covered by PdO. The XPS changes were repeated periodically during the catalytic process, suggesting that different types of palladium ion or oxide, as confirmed *via* TEM analysis (Fig. 3), had a role in improving the catalytic activity. Pd(200) and Pd(200)/PdO(110, 102) facets were formed during catalysis, which demonstrated the individual roles of both Pd(200), as a type of active site, and the Pd(200)/PdO(110, 102) interface, which also played a great role in catalysis due to electron transfer between Pd and PdO.^92–94^

**Fig. 13:**
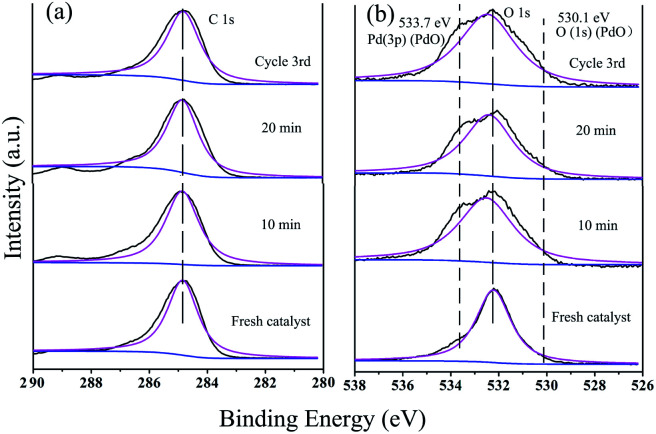
(a) C 1s and (b) O 1s HR-XPS spectra during the catalytic process: fresh catalyst (GO@PDD–Pd); after 10 min; after 20 min; and after the 3^rd^ cycle.

The peak at 284.8 eV in the C 1s spectrum showed no changes with an increase in the reaction time or during recycling, indicating the stability of GO@PDD–Pd (Fig. 13a). Evidence for the existence of PdO could be inferred from the O 1s HR-XPS spectrum due to the exposure of Pd to air (Fig. 13b), and the weak peak appearing at 533.7 eV before catalysis due to Pd 3p_3/2_ (PdO) gradually became more distinct with an increase in time, suggesting the partial oxidation of Pd.^95^ The O 1s peak with a BE of 530.1 eV was assigned to the lattice oxygen of PdO, confirming the existence of PdO during the catalytic process.^79,96^ The presence of both Pd and Pd/PdO in GO@PDD–Pd corroborates the TEM analysis results.

**Scheme 2:**
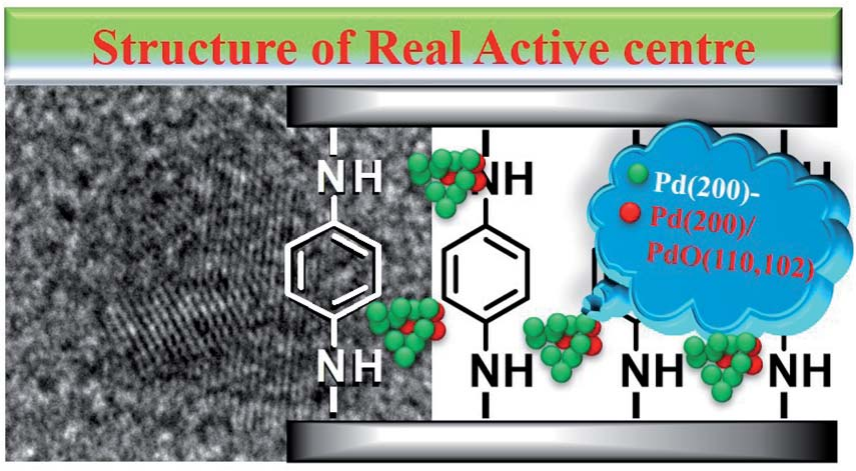
The proposed structure of the formed active centres.

Based on the above results, we speculated that GO as a carrier could transfer electrons to the ligand, and these could be further transferred to Pd^0^, making Pd^0^ more negative. In addition, the Pd(200)/PdO(110, 102) interface generated *in situ* also actively participated in the coupling reaction synergistic to improve the activity,^97^ and the real active centre could be described (Scheme 2).

**Fig. 14:**
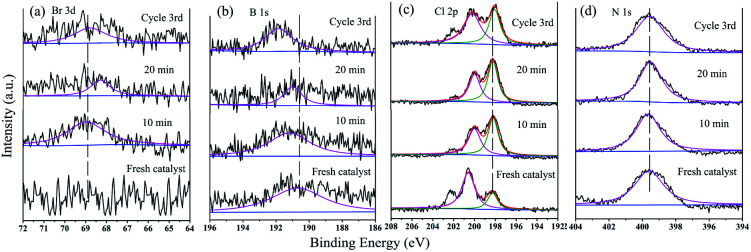
(a) Br 3d, (b) B 1s, (c) Cl 2p, (d) N 1s HR-XPS spectra during the catalytic process and after recycling: 0 min, 10 min, 20 min, and after the 3^rd^ cycle.

Changes in the N 1s, Br 3d, Cl 2p, and B 1s XPS spectra during the catalytic process could further explain the catalytic mechanism (Fig. 14). There was no Br 3d peak in the fresh GO@PPD–Pd spectrum; then, the peak at 68.8 eV exhibited an increasing trend at first, weakening with the progression of the reaction (Fig. 14a), indicating the adsorption of bromo-toluene on the catalyst surface. In addition, a B 1s peak at 190.5 eV appeared and shifted to a higher BE during the catalytic reaction (Fig. 14b), suggesting the adsorption of boronic acid, which reacted with an oxidative addition intermediate to yield a metal translation compound. The peaks at 198.07 eV and 199.81 eV in the Cl 2p spectra showed almost no shift during catalysis and after reuse (Fig. 14c), suggesting that Cl coordinated with Pd^2+^ and Pd atoms during the catalytic process.

The change in the binding energy (BE) of the N 1s peak, which shifted a little from 399.3 eV to 399.7 eV and 399.5 eV during the catalytic process (Fig. 14d), may be related to the loss of electrons. All the changes listed above suggest that the catalytic process proceeded *via* substrate adsorption, intermediate formation, and product desorption.

#### 3.4.4 ReactIR analysis

**Fig. 15:**
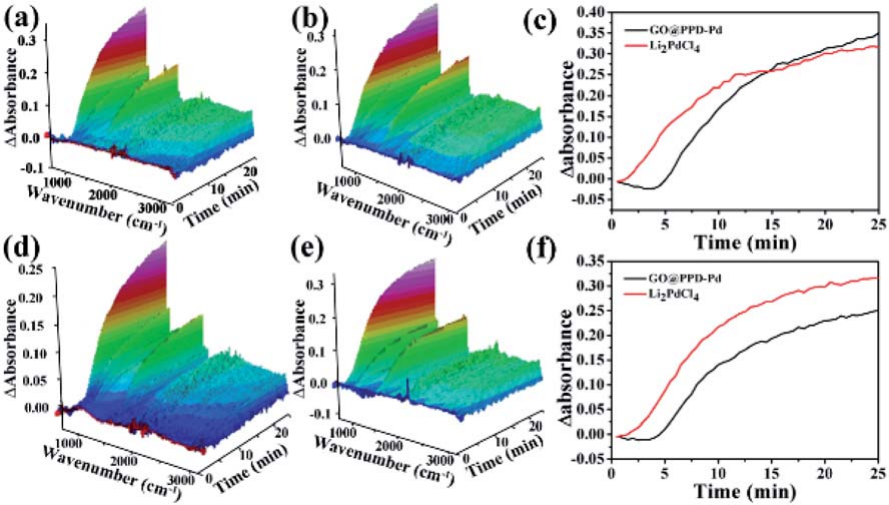
ReactIR plots as a function of the catalytic reaction time during the formation of 4-phenyltoluene at 60 °C: (a) a 3D map obtained using GO@PPD–Pd, (b) a 3D map obtained using Li_2_PdCl_4_, (c) kinetics curves plotted based on GO@PPD–Pd and Li_2_PdCl_4_ at 754 cm^−1^. ReactIR plots as a function of the catalytic reaction time during the formation of 4-phenyltoluene at 40 °C: (d) a 3D map plotted using GO@PPD–Pd as the catalyst, (e) a 3D map obtained using Li_2_PdCl_4_, and (f) kinetics curves plotted based on GO@PPD–Pd and Li_2_PdCl_4_ at 754 cm^−1^.

ReactIR has often been used to monitor reaction processes to elucidate catalytic mechanisms.^98,99^ ReactIR 3D maps from catalysis by GO@PPD–Pd (Fig. 15a) and homogeneous Li_2_PdCl_4_ (Fig. 15b) show differences, indicating the existence of different catalytic mechanisms. The catalytic process curve was measured based on the intensity at 754 cm^−1^, which is designated as the vibration of *para*-substituted benzene (product). For GO@PPD–Pd (Fig. 15c, black line), the peak at 754 cm^−1^ was not detected before 4 min, which was called the “induction period”. Then the intensity increased as time increased. In the case of Li_2_PdCl_4_ (Fig. 15c, red line), a sharp increase was observed in the early stage. This phenomenon arose because Li_2_PdCl_4_ could be evenly dissolved in solution, allowing the substrate to contact with active species easily and generate intermediates rapidly, indicating a different catalytic mechanism. Based on this, a heterogeneous surface catalytic mechanism in the case of GO@PPD–Pd was suggested: the reactants absorbed on the catalyst surface and contacted with active centres to generate intermediates, followed by reacting with aryl boronic acid to yield the product, which diffused into solution upon desorption from the surface. Therefore, the product peak could not be detected at the beginning. Similar results were also obtained at low temperature (Fig. 15d–f).

**Fig. 16:**
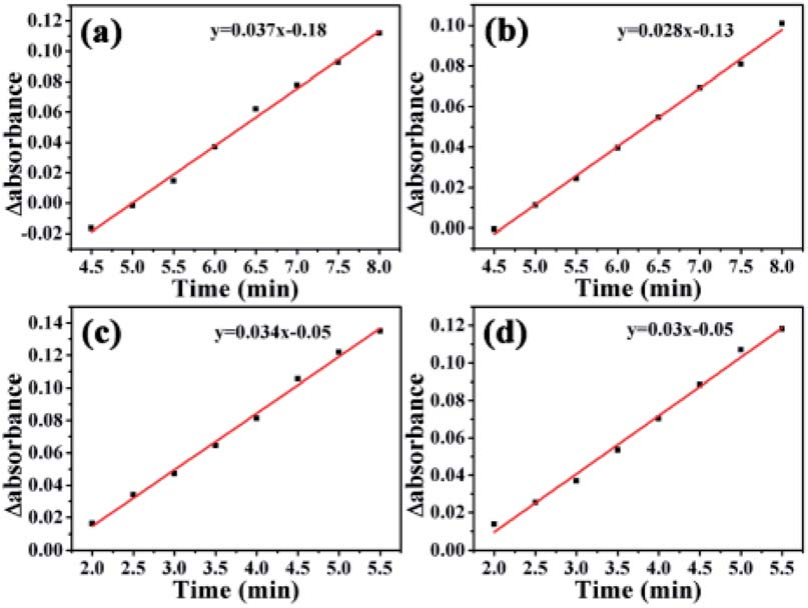
ReactIR-based plots over time during the formation of 4-phenyltoluene *via* the Suzuki coupling reaction at 60 °C: kinetics analysis of the catalytic reaction (a) involving GO@PPD–Pd using the band at 754 cm^−1^ and (c) that catalyzed by Li_2_PdCl_4_. ReactIR plots over time during the formation of 4-phenyltoluene *via* the Suzuki coupling reaction at 40 °C: kinetics analysis of the catalytic reaction (b) involving GO@PPD–Pd using the band at 754 cm^−1^ and (d) that catalyzed by Li_2_PdCl_4_.

The activation energies of GO@PPD–Pd and Li_2_PdCl_4_ were calculated (Fig. 16), and the rate constants (60 °C, *k*_1_ = 0.037; 40 °C, *k*_2_ = 0.028) and activation energy (E_a Hetero_ = 12.1 kJ mol^−1^) of GO@PPD–Pd were obtained. Also, the rate constants (60 °C, *k*_1_ = 0.034; 40 °C, *k*_2_ = 0.03) and activation energy (*E*_a Homo_ = 5.4 kJ mol^−1^) of Li_2_PdCl_4_ were calculated.

**Fig. 17:**
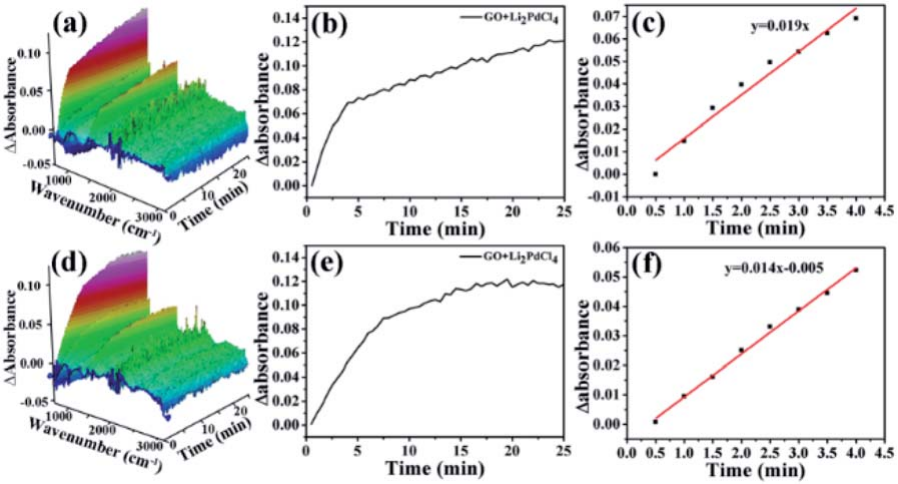
Plots over time during the formation of 4-phenyltoluene: (a) a 3D map (Li_2_PdCl_4_ + GO) at 60 °C, and (b and c) kinetics analysis of the Li_2_PdCl_4_ + GO reaction at 754 cm^−1^; (d) a 3D map (Li_2_PdCl_4_ + GO) at 40 °C, and (e and f) kinetics analysis of Li_2_PdCl_4_ + GO at 754 cm^−1^.

The *E*_a_ results are contrary to theory, because the value of *E*_a Hetero_ was higher than that of *E*_a Homo_. To consider the effects of GO@PPD–Pd dispersion, the same amount of GO was put into the Li_2_PdCl_4_ homogeneous system, and the rate constants (60 °C, *k*_1_ = 0.019; 40 °C, *k*_2_ = 0.014) and *E*_a Homo_ (13.2 kJ mol^−1^) were obtained (Fig. 17). The experimental results showed that *E*_a Homo_ (13.2 kJ mol^−1^) was larger than *E*_a Hetero_ (12.1 kJ mol^−1^), indicating that support dispersion could influence the catalytic activity.

**Scheme 3:**
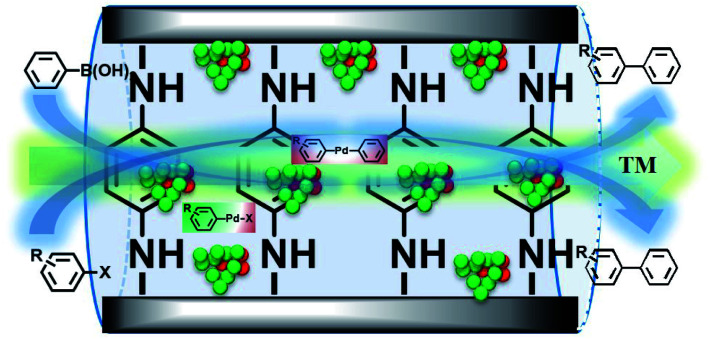
A plausible catalytic mechanism for the Suzuki coupling reaction catalyzed by GO@PPD–Pd.

From all the results listed above, a catalytic mechanism could be described (Scheme 3). At the beginning, the aryl halide reactant absorbed on the surface of the catalytic monolayer where it then contacted with active Pd(200) and the Pd(200)/Pd(110, 102) interface to give oxidative addition intermediates. This was followed by a reaction with phenylboronic acid absorbed on vicinal PdO to yield the intermediate *via* transmetallation, which was then transformed into the product *via* reductive elimination, giving the target molecule (TM, the cross-coupling compound) and releasing Pd(0).

## 4. Conclusions

A catalyst consisting of well-dispersed Pd–Pd/PdO nanoparticles supported on graphene oxide modified with diaminobenzene (GO@PPD–Pd) was prepared, in which graphene oxide was intercalated with PPD as a well-defined support, stabilizing the Pd–Pd/PdO nanoparticles. This was due to strong metal–ligand–support interactions between the amine-functionalized support and nanoparticles. GO@PPD–Pd exhibited high catalytic activity and recyclability. Its deactivation was mainly attributed to (i) the destruction of active centres, including changes to Pd facets and Pd/PdO, and (ii) the aggregation and loss of active species during recycling. The catalytic mechanism involved heterogeneous catalysis occurring at the interface, and the catalytic process involved substrate adsorption, the formation of intermediates, product formation, and product desorption from the catalytic surface. The real active sites were composed of Pd(200) and Pd(200)/PdO(102, 110) with high catalytic activity, and Pd was made more negative *via* the transfer of electrons from the carrier and ligands; also, there was active electron transfer at the interface of Pd/PdO between Pd and PdO. Therefore, the active centres could effectively promote oxidative addition reactions, while Pd(200) and the Pd/PdO interface generated *in situ* also participated in intermediate formation *via* a synergistic effect.

## Conflicts of interest

There are no conflicts to declare.

## Supplementary Material

RA-012-D2RA00658H-s001
