# Supplementary material for: Pd–Pd/PdO as active sites on intercalated graphene oxide modified by diaminobenzene: fabrication, catalysis properties, synergistic effects, and catalytic mechanism
Source: RSC Adv. 2022 Mar 18;12(14):8600–10. doi: 10.1039/d2ra00658h (PMC8984910; doi:10.1039/d2ra00658h)
Supplement: RA-012-D2RA00658H-s001 [file RA-012-D2RA00658H-s001.pdf]

Electronic Supplementary Material (ESI) for RSC Advances.
This journal is © The Royal Society of Chemistry 2022

Supporting Information

Pd-Pd/PdO as Active Sites on the Intercalated Graphene Oxide Modified by Aryl Diamino: Fabrication, Catalysis Property, Synergistic and Catalytic Mechanism

Zihan Li[a], Erran Song[a], Ruirui Ren[a], Wuduo Zhao[a], Tiesheng Li[a*], Minghua Liu[b*] ,Yangjie Wu[a]

[a] *College of Chemistry, Zhengzhou University, Zhengzhou 450001, Henan Province, PR China*

[b] *Beijing National Laboratory for Molecular Science, Institute of Chemistry, Chinese Academy of Sciences, Beijing 100190, PR China*

Contents

1. **Figure captions--2**
2. **Table lists--5**
3. **Additive--9**

1. Figure caption

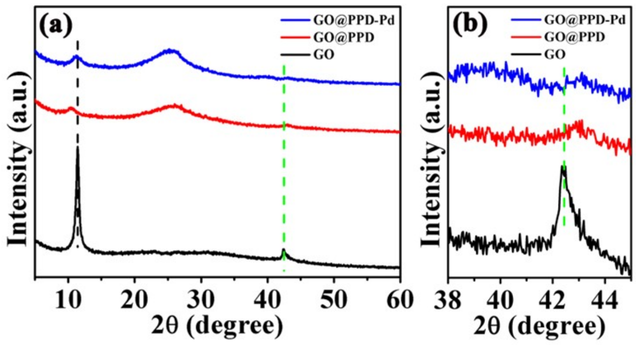

Figure S1 XRD patterns of **GO**, **GO@PPD** and **GO@PPD-Pd**.

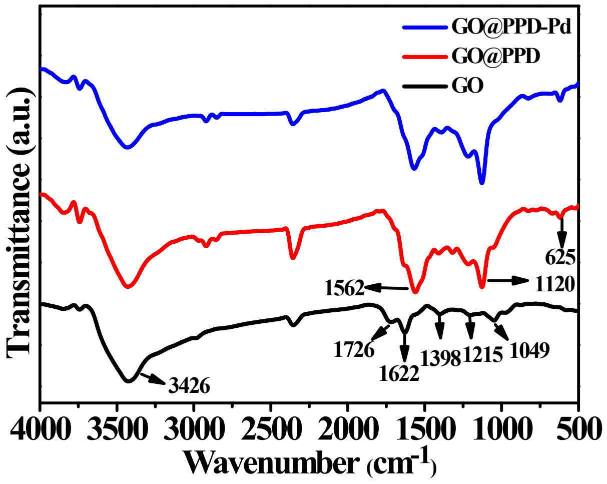

Figure S2 FT-IR spectra of **GO**, **GO@PPD** and **GO@PPD-Pd**.

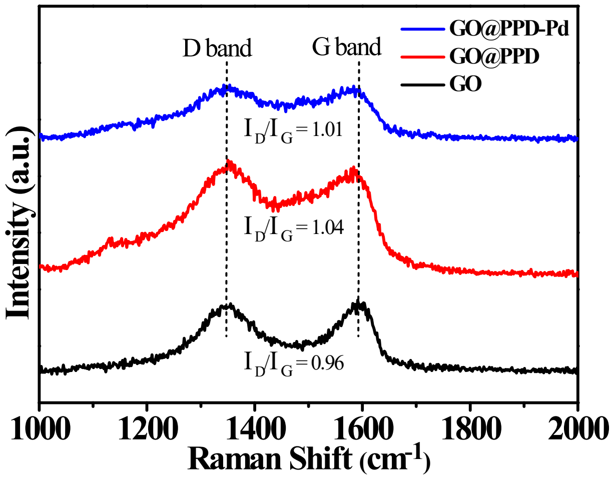

Figure S3. Raman spectrum of **GO**, **GO@PPD** and **GO@PPD-Pd**.

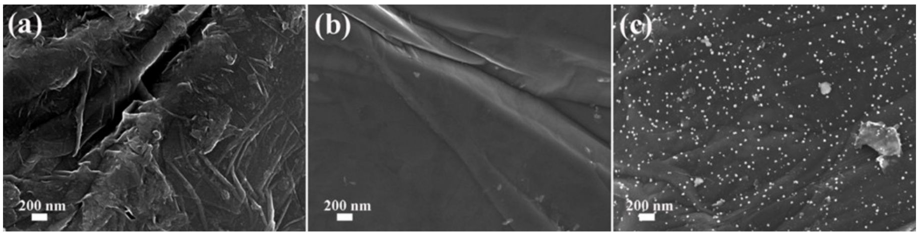

Figure S4 SEM images: (a) **GO**, (b) **GO@PPD**, and (c) **GO@PPD-Pd**.

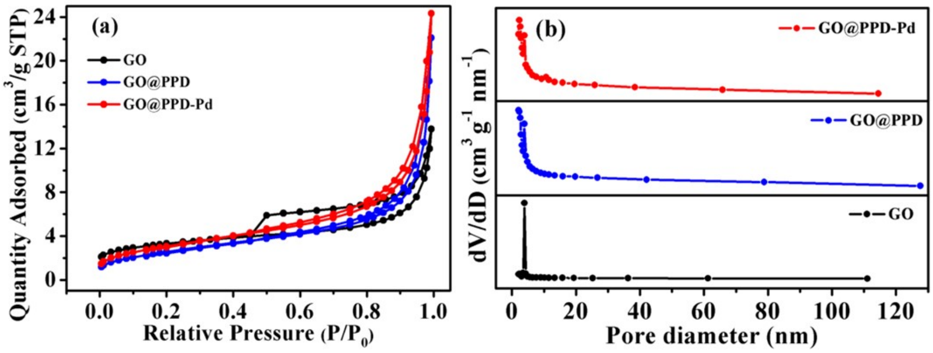

Figure S5 (a) N_2 adsorption-desorption isotherms, (b) the corresponding distribution of pore size for **GO**, **GO@PPD** and **GO@PPD-Pd**, respectively.

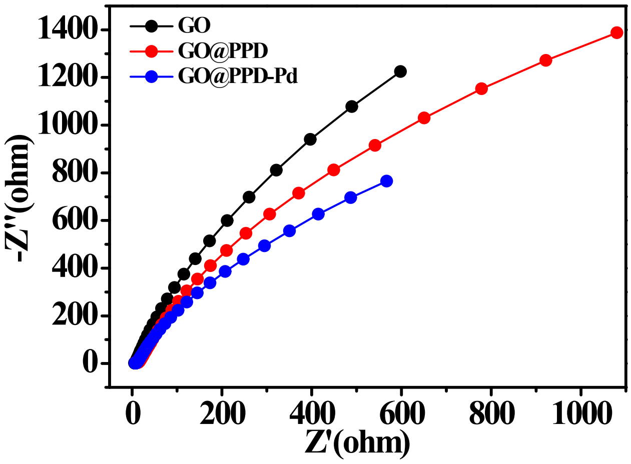

Figure S6 Electrochemical impedance spectra (EIS) of the catalyst on the Ni foam for **GO**, **GO@PPD** and **GO@PPD-Pd**.

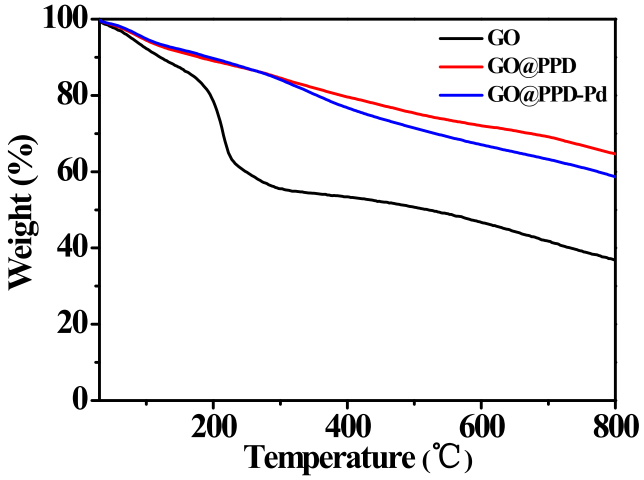

Figure S7 Thermogravimetric analysis curves of **GO**, **GO@PPD** and **GO@PPD-Pd**.

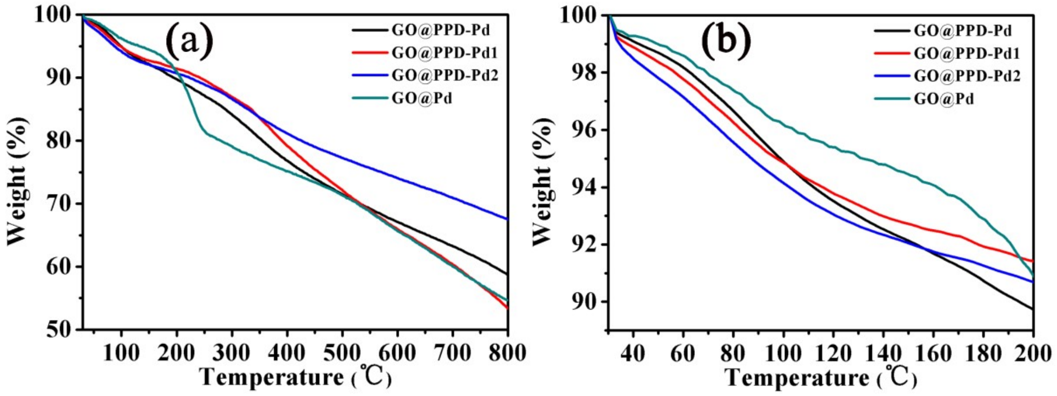

Figure S8 (a)Thermogravimetric analysis curves of **GO-PPD-Pd**, **GO@PPD-Pd1**, **GO@PPD-Pd2** and **GO@Pd**; (b) Amplified section from (a) in the range from 0 to 200 °C.

3. Table List

Table S1. BET surface areas, pore diameter and pore volumes of the catalysts.

Catalyst	S_{BET} (m^2g^{-1})	Pore diameter (nm)	Pore volume (cm^3g^{-1})
**GO**	11.4524	7.7581	0.021552
**GO@PPD**	9.5936	12.8325	0.035923
**GO@PPD-Pd**	11.3250	12.1393	0.039389

Table S2. Optimized conditions in Suzuki coupling reaction by **GO@PPD-Pd**.[a]

Entry	Base	Solvent	Time (min)	T (℃)	Yield (%)[b]	TON[e]	TOF (h^{-1})
**1**	K_2CO_3	H_2O	30	70	13	765	1530
**2**	K_2CO_3	MeOH	30	70	53	3118	6236
**3**	K_2CO_3	EtOH	30	70	91	5353	10706
**4**	K_2CO_3	H_2O:EtOH(5:1)	30	70	62	3647	7294
**5**	K_2CO_3	H_2O:EtOH(4:1)	30	70	67	3941	7882
**6**	K_2CO_3	H_2O:EtOH(3:1)	30	70	76	4471	8942
**7**	K_2CO_3	H_2O:EtOH(2:1)	30	70	89	5235	10470
**8**	K_2CO_3	H_2O:EtOH(1:1)	30	70	99	5824	11648
**9**	K_2CO_3	H_2O:EtOH(1:2)	30	70	92	5412	10824
**10**	K_2CO_3	H_2O:EtOH(1:3)	30	70	83	4882	9764
**11**	Na_2CO_3	H_2O:EtOH(1:1)	30	70	91	5353	10706
**12**	K_3PO_4	H_2O:EtOH(1:1)	30	70	79	4647	9294
**13**	NaOH	H_2O:EtOH(1:1)	30	70	94	5530	11060
**14**	NaOAc	H_2O:EtOH(1:1)	30	70	92	5412	10824
**15**	$NaHCO_3$	H_2O:EtOH(1:1)	30	70	85	5000	10000
**16**	K_2CO_3	H_2O:EtOH(1:1)	30	60	98	5765	11530
**17**	K_2CO_3	H_2O:EtOH(1:1)	30	50	94	5530	11060
**18**	K_2CO_3	H_2O:EtOH(1:1)	30	40	86	5059	10118
**19**	K_2CO_3	H_2O:EtOH(1:1)	30	30	76	4471	8942
**20**	K_2CO_3	H_2O:EtOH(1:1)	20	60	97	5706	17118

**21**	K_2CO_3	H_2O:EtOH(1:1)	10	60	90	5294	31764
**22**	K_2CO_3	H_2O:EtOH(1:1)	20	60	91[c]	10706	32118
**23**	K_2CO_3	H_2O:EtOH(1:1)	20	60	86[d]	20235	60705

[a] Reaction condition: *p*-bromotoluene (0.25 mmol), $PhB(OH)_2$ (0.3 mmol), Base (0.5 mmol), **GO@PPD-Pd** (1 mg), solvent (4 mL). [b] isolated yield. [c] 4-bromotoluene (0.5 mmol), $PhB(OH)_2$ (0.6 mmol), K_2CO_3 (1 mmol). [d] *p*-bromotoluene (1 mmol), $PhB(OH)_2$ (1.2 mmol), K_2CO_3 (2 mmol). [e] 1 mg **GO@PPD-Pd** containing 0.0000425 mmol Pd.

Table S3. Screening substrates of aryl halides with different aryl-boronic acid.[a]

Entry	Ar-X	Ar-B(OH)$_2$	Product	Yield (%)[b]
**1**	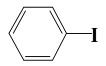	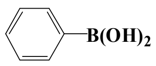	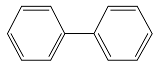	>99
**2**	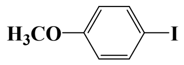	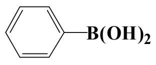	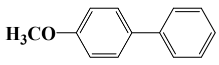	>99
**3**	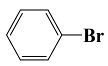	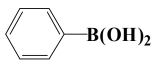	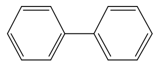	99
**4**	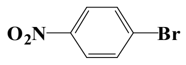	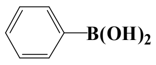	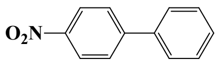	99
**5**	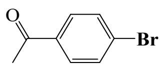	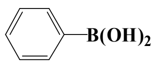	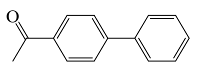	99
**6**	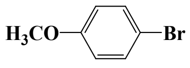	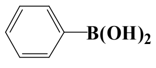	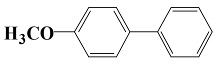	99
**7**	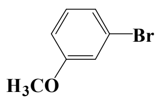	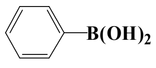	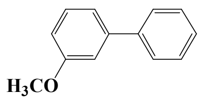	99
**8**	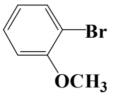	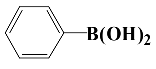	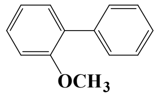	89
**9**	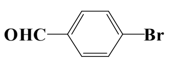	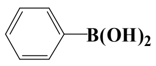	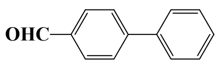	99
**10**	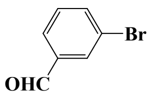	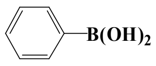	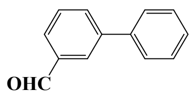	94
**11**	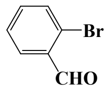	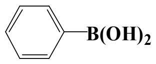	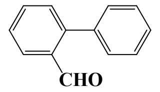	70
**12**	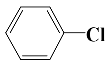	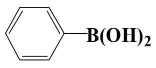	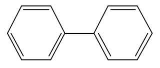	trace
**13**	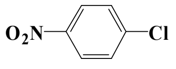	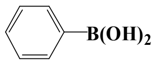	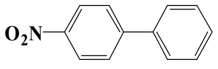	trace

**14**	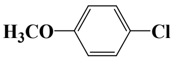	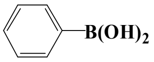	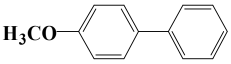	trace

[a] Reaction condition: 4-bromotoluene (0.25 mmol), $PhB(OH)_2$ (0.3 mmol), K_2CO_3 (0.5 mmol), **GO@PPD-Pd** (1 mg), solvent (50% aqueous alcohol 4 mL) at 60 ℃ for 20 min. [b] Isolated yield.

Table S4. Catalytic activity of **GO@PPD-Pd**, **GO@PPD-Pd1**, **GO@PPD-Pd2** and **GO@Pd** for Suzuki-Miyaura reaction.[a]

Catalyst	Base	Solvent	Pd loading ($mmol·mg^{-1}$)	Yield (%)[b]	TON	TOF (h^{-1})
**GO@PPD-Pd**	K_2CO_3	H_2O:EtOH(1:1)	4.25×10^{-5}	97	5706	17118
**GO@PPD-Pd1**	K_2CO_3	H_2O:EtOH(1:1)	1.15×10^{-5}	85	18478	55434
**GO@PPD-Pd2**	K_2CO_3	H_2O:EtOH(1:1)	1.41×10^{-4}	99	1755	5265
**GO@Pd**	K_2CO_3	H_2O:EtOH(1:1)	2.92×10^{-6}	5	4281	12843

[a] Reaction condition: 4-bromotoluene (0.25 mmol), $PhB(OH)_2$ (0.3 mmol), K_2CO_3 (0.5 mmol), catalyst 1 mg, solvent (50% aqueous alcohol 4 mL) at 60 ℃ for 20 min. [b] Isolated yield.

Table S5. Influences of support, ligand and the designed functional catalysts on catalytic properties.[a]

Entry	Catalyst	Pd loading (mmol)	Yield (%)[b]	TON	TOF (h^{-1})
**1**	GO	0	0[c]	0	0
**2**	PPD	0	0[d]	0	0
**3**	GO@PPD	0	0[e]	0	0
**4**	Li_2PdCl_4	0.0000425	76[f]	4471	13413
**5**	GO+Li_2PdCl_4	0.0000425	64[g]	3765	11295
**6**	PPD+Li_2PdCl_4	0.0000425	trace[h]	-	-
**7**	GO@PPD+Li_2PdCl_4	0.0000425	38[i]	2235	6705
**8**	GO@PPD-Pd	0.0000425	97[j]	5706	17118
**9**	Si@PPD-Pd	0.00025	90[k]	900	2700

[a] Reaction condition: 4-bromotoluene (0.25 mmol), $PhB(OH)_2$ (0.3 mmol), K_2CO_3 (0.5 mmol), solvent (50% aqueous alcohol 4 mL) at 60 ℃ for 20 min. [b] Isolated yield. [c] GO 1 mg, [d] PPD 1 mg, [e] GO@PPD 1 mg, [f] Li_2PdCl_4 0.0000425 mmol, [g] GO 1 mg and Li_2PdCl_4 0.0000425 mmol, [h] PPD 1 mg and Li_2PdCl_4 0.0000425 mmol, [i] GO@PPD 1 mg and Li_2PdCl_4 0.0000425 mmol, [j] GO@PPD-Pd 1 mg containing 0.0000425 mmol Pd, [k] Si@PPD-Pd 1 mg containing 0.00025 mmol Pd.

Table 6. Influences of catalysts prepared with different ligands used to modified GO on catalytic performance.

**Entry**	**Ligand**	**Pd loading ($mmol·mg^{-1}$)**	**yield (%)**	**TON**	**TOF(h^{-1})**
**1**	*p*-phenylenediamine	4.25×10^{-5}	97	5706	17118
**2**	*m*-phenylenediamine	1.57×10^{-4}	96	1529	4587
**3**	*o*-phenylenediamine	1.12×10^{-5}	95	21205	63615
**4**	*p*-aminotoluene	6.51×10^{-5}	98	3764	11292
**5**	*p*-nitroaniline	1.74×10^{-4}	96	1379	4137
**6**	phenylamine	8.15×10^{-5}	97	2976	8928
**7**	1,3-diaminopropane	8.57×10^{-5}	84	2451	7353
**8**	1,6-diaminohexane	6.04×10^{-5}	75	3104	9312

[a] Reaction condition: 4-bromotoluene (0.25 mmol), $PhB(OH)_2$ (0.3 mmol), K_2CO_3 (0.5 mmol), catalyst 1 mg, solvent (50% aqueous alcohol 4 mL) at 60 °C for 20 min.

Table S7. Comparison of the similar Pd catalysts reported.

**Entry**	**Catalyst**	**Reaction conditions**	**X**	**Yield (%)**	**TOF (h^{-1})**	**Ref**
1	GO@PPD-Pd	K_2CO_3, H_2O:EtOH (1:1), 20 min, 60 ℃	4-bromotoluene	97	17118	This work
2	ASNTs@Pd	K_2CO_3, EtOH, 30 min, 80 ℃	Bromobenzene	99	3444	24
3	Pd/PRGO	K_2CO_3, H_2O:EtOH (1:1), 5 min, 120 ℃	Bromobenzene	100	23000	54
4	F-GO-Pd	K_2CO_3, H_2O:EtOH (1:1), 20 min, 70 ℃	Bromobenzene	97	21008	78
5	Pd@APGO	K_2CO_3, H_2O:EtOH (1:1), 6 h, 80 ℃	Iodobenzene	96	68	55
6	Pd@GOF	K_2CO_3, Toluene, 6 h, 80 ℃	Bromobenzene	>99	8	63
7	Pd/RGO-0.025 PPD	K_2CO_3, H_2O:EtOH (1:1), 15min, rt （K_2CO_3, H_2O:EtOH (1:1), 2min, under MWI at 80 ℃）	Bromobenzene	95 (96.7)	1740 (96,700)	52

**8**	DNA-G-Pd (1.1 mol %)	K_3PO_4, H_2O, SDS, 4min, 100 ℃	Iodobrnzene	100	1363	56
**9**	GO-NH_2-Pd^{2+} (1.0 mol %)	K_2CO_3,EtOH/H_2O(2:1), 4 h, 60 ℃	Bromobenzene	73	1825	20

Table S8. Poisoning experiments of **GO@PPD-Pd** catalyst.[a]

Entry	Poisoning additive	Yield (%)[d]
**1**	-	97
**2**	2,2'-Dipyridyl	5[b]
**3**	Thiophene	31[c]

[a] Reaction condition: 4-bromotoluene (0.25 mmol), $PhB(OH)_2$ (0.3 mmol), K_2CO_3 (0.5 mmol), **GO@PPD-Pd** 1 mg, solvent (4 mL) at 60 ℃ for 20 min. [b] 0.5 equiv of 2,2'-Dipyridyl (per metal atom). [c] 0.5 equiv of Thiophene (per metal atom). [d] Isolated yield.

Additive: Characterization of coupling compounds in Suzuki coupling reaction

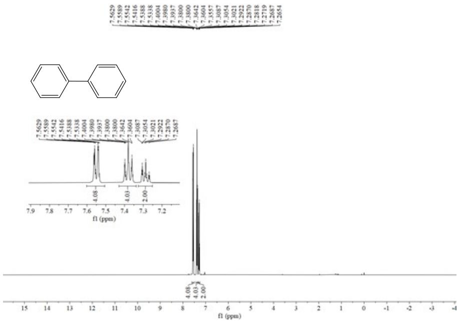

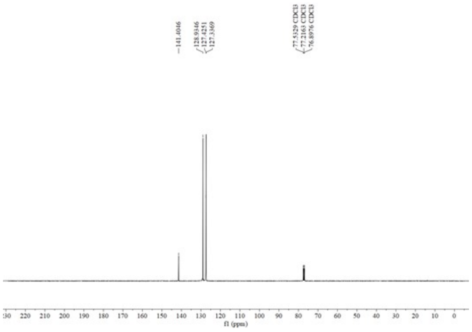

Entry 1: ^1H NMR (400 MHz, $CDCl_3$) δ 7.55 (dt, J = 8.2, 1.8 Hz, 4H), 7.43 – 7.34 (m, 4H), 7.33 – 7.25 (m, 2H).; ^{13}C NMR (101 MHz, $CDCl_3$) δ 141.4, 128.9, 127.4, 127.3.

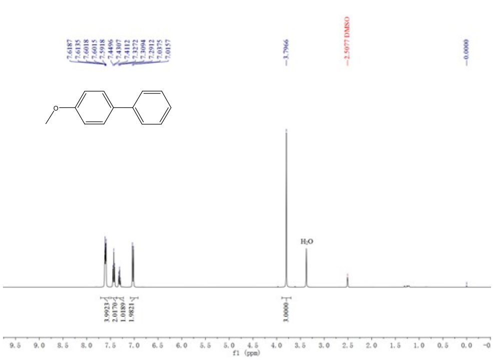

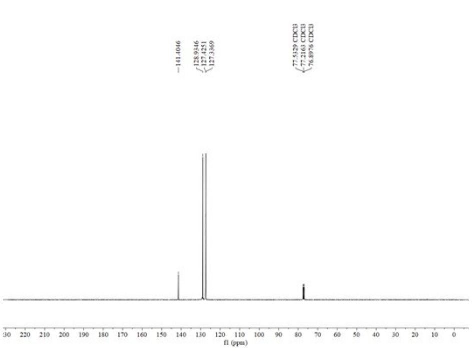

Entry 2. ^1H NMR (400MHz, DMSO, δ ppm): 7.47-7.45(m, 2H), 7.41-7.38(m, 2H), 7.30-7.36(m,

2H), 7.26-7.29(dd, J2=1.8 Hz, J1=7.52 Hz,1H), 7.11-7.09(d,J=8.28 Hz, 1H), 7.02(m, 1H), 3.75(s, 3H).; ^{13}C NMR (101 MHz, $CDCl_3$, δ ppm) :159.28, 140.94, 133.82, 128.84, 128.25, 126.81 (d, J = 7.1 Hz), 114.31, 55.43.

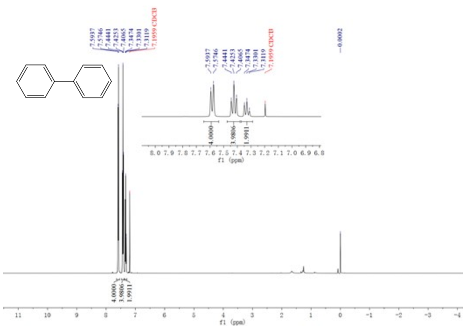

Entry 3： ^{1}H NMR(400MHz, DMSO, δ ppm)**:** 7.59-757(d, J=7.64 Hz,4H), 7.44-7.40(t, J=7.52, 4H), 7.34-7.31(t, J=6.92 Hz, 2H). ; ^{13}C NMR (101 MHz, $CDCl_3$) δ 141.4, 128.9, 127.4, 127.3.

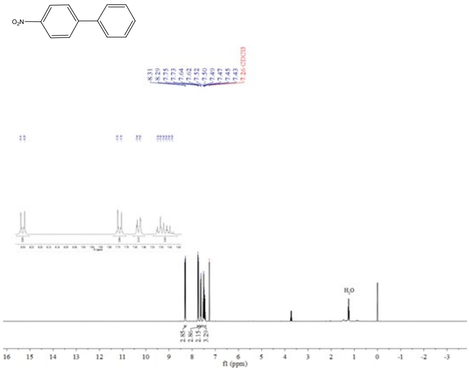

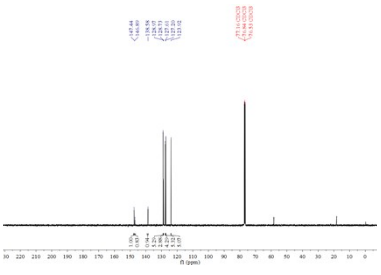

Entry 4. 1H NMR (400 MHz, CDCl3，δ ppm): 7.41 – 7.55 (m, 3H),7.63 (d, J = 6.8 Hz, 2H),7.74 (d, J = 8.8 Hz, 2H),8.30 (d, J = 8.8 Hz, 2H).; ^{13}C NMR (101 MHz, $CDCl_3$, δ ppm): 147.44 (1C), 146.89 (1C), 138.58 (1C), 128.97 (2C), 128.73 (1C), 127.61 (2C), 127.20 (2C), 123.92 (2C).

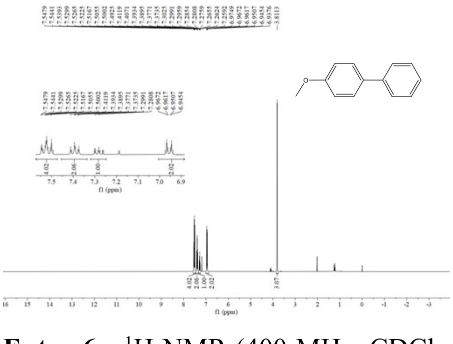

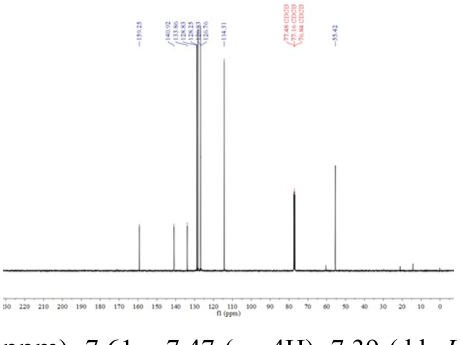

Entry 6： ^{1}H NMR (400 MHz, $CDCl_3$, δ ppm) :7.61 – 7.47 (m, 4H), 7.39 (dd, J = 8.5, 6.9 Hz, 2H), 7.32 – 7.24 (m, 1H), 7.00 – 6.92 (m, 2H), 3.81 (s, 3H).; ^{13}C NMR (101 MHz, $CDCl_3$, δ ppm) :159.25, 140.92, 133.86, 128.83, 128.25, 126.80, 114.31, 55.42.

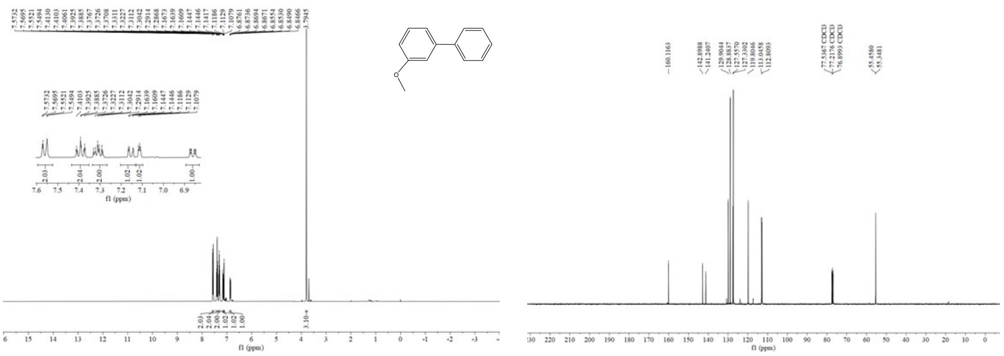

Entry 7: ^{1}H NMR (400 MHz, $CDCl_3$) δ 7.56 (dd, *J* = 8.2, 1.3 Hz, 2H), 7.39 (dd, *J* = 8.3, 6.7 Hz, 2H), 7.34 – 7.26 (m, 2H), 7.20 – 7.13 (m, 1H), 7.12 – 7.10 (m, 1H), 6.86 (ddd, *J* = 8.2, 2.6, 1.0 Hz, 1H), 3.79 (s, 3H).; ^{13}C NMR (101 MHz, $CDCl_3$) δ 160.1, 142.9, 141.2, 129.9, 128.9, 127.6, 127.3, 119.8, 113.0, 112.8, 55.3.

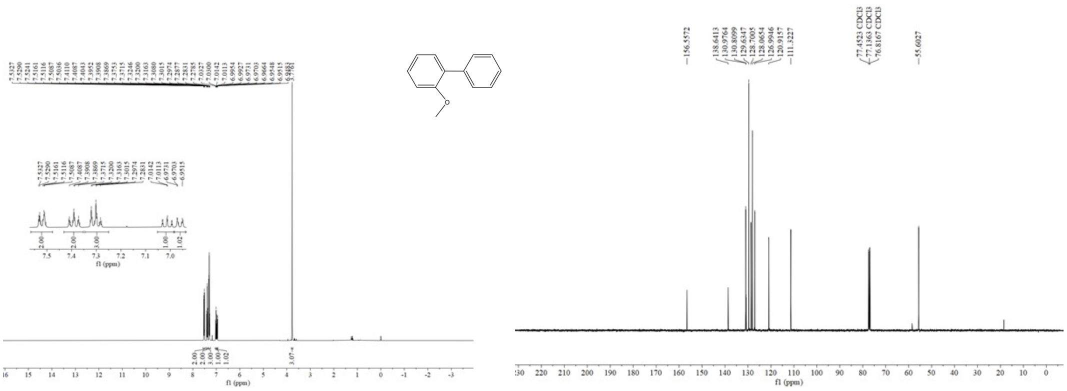

Entry 8: ^{1}H NMR (400 MHz, $CDCl_3$) δ 7.56 – 7.48 (m, 2H), 7.43 – 7.34 (m, 2H), 7.30 (td, *J* = 7.5, 1.6 Hz, 3H), 7.06 – 6.93 (m, 2H), 3.78 (s, 3H).; ^{13}C NMR (101 MHz, $CDCl_3$) δ 156.6, 138.6, 131.0, 130.8, 129.6, 128.7, 128.1, 127.0, 120.9, 111.3, 55.6.

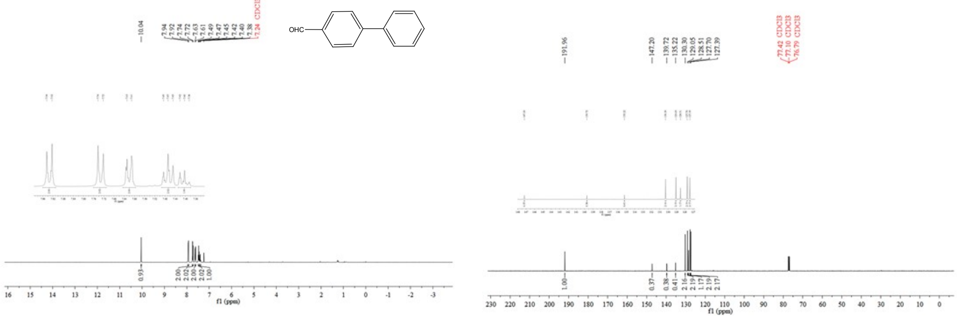

Entry 9: ^{1}H NMR (400 MHz, $CDCl_3$, δ ppm): 10.04 (s, 1H), 7.93 (d, *J* = 8.3 Hz, 2H), 7.73 (d, *J* = 8.3 Hz, 2H), 7.62 (d, *J* = 7.1 Hz, 2H), 7.47 (t, *J* = 7.4 Hz, 2H), 7.40 (t, *J* = 7.3 Hz, 1H).;^{13}C NMR (101 MHz, $CDCl_3$, δ ppm): 191.96, 147.20, 139.72, 135.22, 130.30, 129.05, 128.51, 127.70, 127.39.

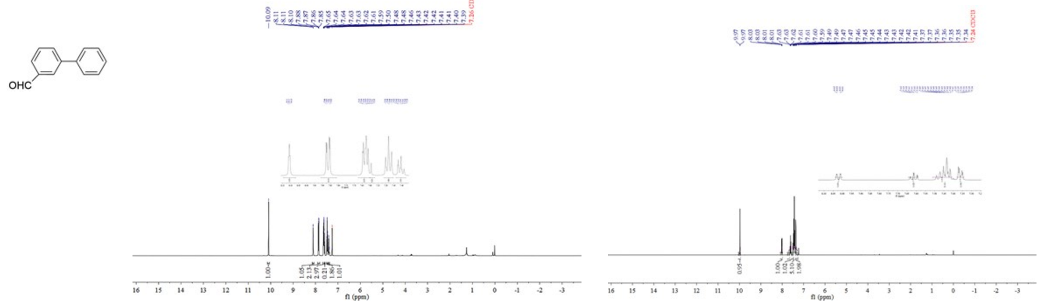

Entry 10: ^{1}H NMR (400 MHz, $CDCl_3$, δ ppm) : 10.09 (s, 1H), 8.11 (dt, *J* = 1.8 Hz, 1H), 7.86 (dd, *J* = 7.7, 1.8 Hz, 2H), 7.67 (m, 2H), 7.60 (m, 1H), 7.48 (t, *J* = 7.5 Hz, 2H), 7.44 – 7.38 (m, 1H).;^{13}C NMR (101 MHz, CDCl3，δ ppm) :192.42, 142.26, 139.79, 137.03, 133.16, 129.60, 129.11, 128.73, 128.30, 128.12, 127.25.

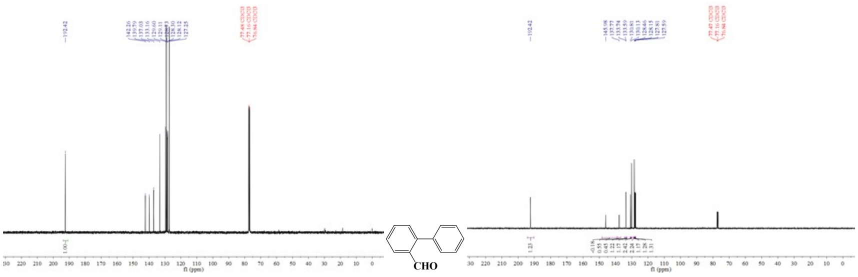

Entry 11: ^{1}H NMR (400 MHz, $CDCl_3$) δ 9.97 (d, *J* = 0.7 Hz, 1H), 8.02 (dd, *J* = 7.8, 1.3 Hz, 1H), 7.64 – 7.59 (m, 1H), 7.51 – 7.41 (m, 5H), 7.38 – 7.34 (m, 2H).;^{13}C NMR (101 MHz, $CDCl_3$, δ ppm): 192.42, 145.98, 137.77, 133.74, 133.59, 130.81, 130.13, 128.46, 128.15, 127.81, 127.59.

Reference

(1) M.L.N. Rao et al. A new palladium catalyzed protocol for atom-efficient cross-coupling reactions of triarylbismuths with aryl halides and triflates. Tetrahedron. 2008; 64: 5762–5772.

(2) Rai RK, Gupta K, Behrens S, Li J, Xu Q, Singh Sanjay K. Highly Active Bimetallic Nickel–Palladium Alloy Nanoparticle Catalyzed Suzuki–Miyaura Reactions. ChemCatChem. 2015;7: 1806 – 1812.

(3) Doustkhah E, Rostamnia S, Imura M. Thiourea bridged periodic mesoporous organosilica with ultra-small Pd nanoparticles for coupling reactions. RSC Adv. 2017; 7: 56306–56310.

(4) Lü B, Fu CL, Ma SM. Application of a readily available and air stable monophosphine HBF4 salt for the Suzuki coupling reaction of aryl or 1-alkenyl chlorides. Tetrahedron Lett. 2010; 51: 1284–1286.

(5) Bao GG, Bai J, Li CP. Synergistic effect of the Pd–Ni bimetal/carbon nanofiber composite catalyst in Suzuki coupling reaction. Org. Chem. Front. 2019; 6: 352–361.

(6)Mino T, Shirae Y, Sakomoto M, Fujita T. Phosphine-Free Hydrazone-Pd Complex as the Catalyst Precursor for a Suzuki-Miyaura Reaction under Mild Aerobic Conditions. J. Org. Chem. 2005, 70, 2191-2194.

(7) Bandini M, Pietrangelo A, Sinisi R, Ronchi AU, Wolf MO. New Electrochemically Generated Polymeric Pd Complexes as Heterogeneous Catalysts for Suzuki Cross-Coupling Reactions. Eur. J.

Org. Chem. 2009; 3554–3561.
